# Higher-Ranked Annotation Polymorphic Dependency Analysis

**DOI:** 10.1007/978-3-030-44914-8_24

**Published:** 2020-04-18

**Authors:** Fabian Thorand, Jurriaan Hage

**Affiliations:** ETH Zurich, Zurich, Switzerland; Department of Information and Computing Sciences, Utrecht University, Utrecht, The Netherlands

## Abstract

The precision of a static analysis can be improved by increasing the context-sensitivity of the analysis. In a type-based formulation of static analysis for functional languages this can be achieved by, e.g., introducing let-polyvariance or subtyping. In this paper we go one step further by defining a higher-ranked polyvariant type system so that even properties of lambda-bound identifiers can be generalized over. We do this for dependency analysis, a generic analysis that can be instantiated to a range of different analyses that in this way all can profit.

We prove that our analysis is sound with respect to a call-by-name semantics and that it satisfies a so-called noninterference property. We provide a type reconstruction algorithm that we have proven to be terminating, and sound and complete with respect to its declarative specification. Our principled description can serve as a blueprint for making other analyses higher-ranked.

## 1 Introduction

The typical compiler for a statically typed functional language will perform a number of analyses for validation, optimisation, or both (e.g., strictness analysis, control-flow analysis, and binding time analysis). These analyses can be specified as a type-based static analysis so that vocabulary, implementation and concepts from the world of type systems can be reused in this setting [19,24]. In that setting the analysis properties are taken from a language of annotations which adorn the types computed for the program during type inference: the analysis is specified as an annotated type system, and the payload of the analysis corresponds to the annotations computed for a given program.

Consider for example binding-time analysis [5,7]. In this case, we have a two-value lattice of annotations containing $S$ for static and $D$ for dynamic (where $\bot = S \sqsubset D = \top$, so that whenever an expression is annotated with $S$, it can be soundly changed to $D$, because that is a strictly weaker property). An expression that is known to be static may be evaluated at compile time, because the analysis has determined that all the values that determine its outcome are in fact available at compile-time while all other expressions are annotated with $D$, and must be evaluated at run-time; the goal of binding-time analysis is then to (soundly) assign $S$ to as many expressions as possible.

Static analyses may differ in precision, e.g., a monovariant binding-time analysis lacks context-sensitivity for let-bound identifiers (although some of it can be recovered with subtyping). Assuming *id* to be the identity function, if in the program

$$\textbf{let}\ id\ x = x\ \textbf{in} \ldots id\ s \ldots id\ d \ldots$$

the subexpression $s$ is a statically known integer, which we denote as $s : \mathrm{int}\langle S\rangle$, and $d : \mathrm{int}\langle D\rangle$ a dynamic integer, then for *id* we arrive at $\mathrm{int}\langle D\rangle \to \mathrm{int}\langle D\rangle$, so that the property found for *id s* is that it is a dynamic integer. Clearly, however, if the value of $s$ is known statically then also that of *id s* is! The fact that values with different properties flow to a function and we have to be (overly) pessimistic for some of these is a phenomenon sometimes called *poisoning* [28]. Context-sensitivity reduces poisoning; it can be achieved by making the analysis *polyvariant*. In that case, our type for *id* may become $\forall\beta.\mathrm{int}\langle\beta\rangle \to \mathrm{int}\langle\beta\rangle$, so that for the first call to *id* we may instantiate $\beta$ with $S$ and for the second choose $D$, essentially mimicking the polymorphic lambda-calculus at the level of annotations.

But what about a function like

$$foo = \lambda f.\,(f\ d, f\ s)$$

in which we have two calls to a lambda-bound function argument $f$? Can we treat these context-sensitively as well, so that we can have the most precise types for both calls, independent of each other? The answer is: yes, we can.

Independence can be achieved by inferring for *foo* a type that associates with $f$ an annotation polymorphic type,

$$\forall\beta_1.(\forall\beta_0.\mathrm{int}\langle\beta_0\rangle \to \mathrm{int}\langle\beta_1\ \beta_0\rangle)$$

Here, $\beta_0$ ranges over simple annotations (such as $S$ and $D$), and $\beta_1$ ranges over annotation level functions (in the terminology of this paper, these annotations are higher-sorted; see section 3). The annotation variable $\beta_0$ is a placeholder for the analysis property of the actual argument to $f$, while $\beta_1$ represents how that property propagates to the value returned by $f$. If the identity function $\forall\beta.\mathrm{int}\langle\beta\rangle \to \mathrm{int}\langle\beta\rangle$ is passed to *foo*, a pair with annotated type $\mathrm{int}\langle D\rangle \times \mathrm{int}\langle S\rangle$ will be returned. This is because the types of $f\ d$ and $f\ s$ can be determined independently of each other, because the choice for $\beta_0$ can be made separately for each call. The "price" we pay is that we have to know how the annotations on the values returned by $f$ can be derived from the annotations on the arguments. This is exactly what $\beta_1$ represents.

If $\beta_0$ or $\beta_1$ would range over (annotated) types, then the underlying language itself would be higher-ranked, and inference in that case is known to be undecidable [14]. However, as we show in this paper, if they range only over annotations (even higher-sorted ones), then inference may become decidable again. Why is that? Intuitively, this is because the underlying types provide structure to the analysis inference algorithm, while a higher-ranked polymorphic type system does not have this advantage.

In which situations can we expect to benefit from higher-ranked polyvariance? Generally speaking, this is when we have functions of order 2 and higher, functions that often show up in idiomatic functional code.

Languages like Haskell do support higher-rank types [13]. Decidability is not problematic then, because the compiler expects the programmer to provide the higher-rank type signatures where necessary, and the compiler only needs to verify that the provided types are consistent: type checking *is* decidable. In our situation this is typically not acceptable: we cannot expect programmers to provide explicit control-flow [12] or binding-time information. So we have to insist on full inference of analysis information, and this paper shows how this can be done for dependency analysis [1].

Dependency analysis is in fact a family of analyses; instances include binding-time analysis, exception analysis, secure information flow analysis and static slicing. The precision of our higher-ranked polyvariant annotated type system for dependency analysis thereby carries over immediately to the instances, and metatheoretical properties we prove, like a noninterference theorem [8], need to be proven only once.

In summary, this paper offers the following *contributions*. We (1) define a higher-ranked annotation polymorphic type system for a generic dependency analysis (section 4) for a call-by-name language that takes its annotations from a simply typed lambda-calculus enriched with lattice operations (section 3). The analysis also supports polyvariant recursion [10] to improve precision for certain recursive functions. Due to the principled way in which the analysis is set-up it can serve as a blueprint for giving other analyses the same treatment. We (2) prove our system sound with respect to a call-by-name operational semantics. We also formulate and prove a noninterference theorem for our system (section 5). We (3) give a type reconstruction algorithm that is sound and complete with respect to the type system (section 6) and provide a prototype implementation (section 7). For reasons of space we omit many details that are available in a separate document [26].

## 2 Intuition and motivation

Before we go on to the technical details of this paper, we want to elaborate upon our intuitive description from the introduction. We do this by means of a few small examples, keeping the discussion informal. Formally discussed examples, as generated by our implementation, become big and hard to read pretty quickly; these can be found in section 7.

We start with a few examples in which binding-time analysis is the dependency analysis instance, followed by a few examples that use security flow analysis; our implementation supports both instances. We note that our implementation supports a few more language constructs than the formal specification given in this paper, giving us a bit more flexibility. Neither, however, supports polymorphism at the type level. This substantially simplifies the technicalities.

For the following example

$$foo : ((\text{int} \to \text{int}) \to \text{int}) \to \text{int} \times \text{int}$$
$$foo = \lambda f : (\text{int} \to \text{int}) \to \text{int}.(f\ (\lambda x : \text{int}.x), f\ (\lambda x : \text{int}.0))$$

our analysis can derive a higher-ranked polyvariant type for $f$,

$$\forall \beta_1.(\forall \beta_2.\text{int}\langle \beta_2 \rangle \to \text{int}\langle \beta_1\ \beta_2 \rangle) \to \text{int}\langle \beta_3\ \beta_2\ \beta_1 \rangle$$

where $\beta_1$ and $\beta_2$ can be instantiated independently for each of the two calls to $f$ in *foo*, and $\beta_3$ is universally bound by *foo* and represents how the argument $f$ uses its function argument.

Since the argument to $f$ is itself a function, the information that flows out of, say, the first call to $f$ can be independent of the analysis of the function that flows into the second call (and vice versa), thereby avoiding unnecessary poisoning. This means that the binding-time of, say, the second component of the pair depends only on $f$ and the function $\lambda x : \text{int}.0$, irrespective of $f$ also receiving $\lambda x : \text{int}.x$ as argument to compute the first component.

For the next example, let us consider security flow analysis in which we have annotations $L$ and $H$ that designate values (call these $L$-values and $H$-values) of low respectively high confidentiality. An important scenario where additional precision can be achieved is when analyzing Haskell code in which type classes have been desugared to dictionary-passing functional core. A function like

$$g\ x\ y = (x + y, y + y)$$

is then transformed into something like $g\ (+)\ x\ y = (x + y, y + y)$. Now, consider the case that we pass an $H$-value to $x$ and an $L$-value to $y$; the operator $(+)$ produces an $L$-value if and only if both arguments are $L$-values. Without higher-ranked annotations, the annotation on the first argument to $(+)$ has to be consistent with all uses of $(+)$. Because $x$ is an $H$-value, that will then also be the case for the second call to $(+)$, leading to a pair of values of which the components are both $H$-values. With higher-ranked annotations, we can instantiate the two instances independently, and the second component of the pair is analyzed to produce an $L$-value. Functions in Haskell that use type classes are extremely common.

## 3 The $\lambda^{\sqcup}$-calculus

An essential ingredient of our annotated type system is the language of annotations that we use to decorate our types and to represent the dependencies resulting from evaluating an expression. Indeed, the fact that annotations are in fact "programs" in a lambda calculus is what allows us to make our analysis a higher-ranked polyvariant one. For the purpose of this paper, we generalize the $\lambda^{\cup}$-calculus of [16] to the $\lambda^{\sqcup}$-calculus ($\lambda^{\sqcup}$ for short) a simply typed lambda calculus extended with a lattice structure.

The syntax of $\lambda^{\sqcup}$ is given in figure 1; from now on, we refer to its types exclusively as *sorts*. Here, $\kappa$ ranges over sorts, $\beta$ over annotation variables, etc.

$$
\begin{array}{llr}
\kappa \in \mathbf{AnnSort} ::= \star & & \text{(base sort)} \\
\quad\quad\quad\quad \mid \kappa_1 \Rightarrow \kappa_2 & & \text{(function sort)} \\
\beta \in \mathbf{AnnVar} & & \text{(annotation variables)} \\
\xi \in \mathbf{AnnTm} ::= \beta & & \text{(variable)} \\
\quad\quad\quad\quad \mid \lambda\beta :: \kappa.\xi & & \text{(abstraction)} \\
\quad\quad\quad\quad \mid \xi_1\ \xi_2 & & \text{(application)} \\
\quad\quad\quad\quad \mid \ell & & \text{(lattice value, } \ell \in \mathcal{L}\text{)} \\
\quad\quad\quad\quad \mid \xi_1 \sqcup \xi_2 & & \text{(lattice join operation)}
\end{array}
$$

Fig. 1: The syntax of the $\lambda^{\sqcup}$-calculus, sorts and annotations

In order to avoid confusion with the field of (algebraic) effects, we refer to terms of $\lambda^{\sqcup}$ as *dependency terms* or *dependency annotations*. Terms are either of base sort $\star$, representing values in the underlying lattice $\mathcal{L}$, or of function sort $\kappa_1 \Rightarrow \kappa_2$.

On the term level, we allow arbitrary elements of the underlying lattice and taking binary joins, in addition to the usual variables, function applications and lambda abstractions. Lattice elements are assumed to be taken from a *bounded join-semilattice* $\mathcal{L}$, an algebraic structure $\langle L, \sqcup \rangle$ consisting of an underlying set $L$ and an associative, commutative and idempotent binary operation $\sqcup$, called *join* (we usually write $\ell \in \mathcal{L}$ for $\ell \in L$), and a least element $\bot$.

The sorting rules of $\lambda^{\sqcup}$ are straightforward (see [26]). Values of the underlying lattice are always of sort $\star$, and the join operator is defined on arbitrary terms of the same sort:

$$\frac{\Sigma \vdash_{\mathrm{s}} \xi_1 : \kappa \qquad \Sigma \vdash_{\mathrm{s}} \xi_2 : \kappa}{\Sigma \vdash_{\mathrm{s}} \xi_1 \sqcup \xi_2 : \kappa}\ [\text{S-JOIN}]$$

The sorting rule uses *sort environments* denoted by the letter $\Sigma$ that map annotation variables $\beta$ to sorts $\kappa$. We denote the set of sort environments by **SortEnv**. More precisely, a *sort environment* or *sort context* $\Sigma$ is a finite list of bindings from annotation variables $\beta$ to sorts $\kappa$. The empty context is written as $\emptyset$ (in code as []), and the context $\Sigma$ extended with the binding of the variable

$$
\begin{aligned}
V_\star &= \mathcal{L} \\
V_{\kappa_1 \Rightarrow \kappa_2} &= \{f : V_{\kappa_1} \to V_{\kappa_2} \mid f \text{ mono}\} \\
\rho : \mathbf{AnnVar} &\to_{\text{fin}} \bigcup \{V_\kappa \mid \kappa \in \mathbf{AnnSort}\} \\
[\![\beta]\!]_\rho &= \rho(\beta) \\
[\![\lambda\beta :: \kappa_1.\xi]\!]_\rho &= \lambda v \in V_{\kappa_1}.\, [\![\xi]\!]_{\rho[\beta \mapsto v]} \\
[\![\xi_1\ \xi_2]\!]_\rho &= [\![\xi_1]\!]_\rho\,([\![\xi_2]\!]_\rho) \\
[\![\ell]\!]_\rho &= \ell \\
[\![\xi_1 \sqcup \xi_2]\!]_\rho &= [\![\xi_1]\!]_\rho \sqcup [\![\xi_2]\!]_\rho
\end{aligned}
$$

Fig. 2: The semantics of $\lambda^{\sqcup}$-calculus

$\beta$ to the sort $\kappa$ is written $\Sigma, \beta : \kappa$. We denote the set of annotation variables in the context $\Sigma$ with $\mathrm{dom}(\Sigma)$. When we write, $\Sigma(\beta) = \kappa$ this means that $\beta \in \mathrm{dom}(\Sigma)$ and the rightmost occurrence of $\beta$ binds it to $\kappa$. Moreover, $\Sigma \setminus B$ where $B \subseteq \mathbf{AnnVar}$ denotes the context $\Sigma$ where all bindings of annotation variables in $B$ have been removed. In the remainder of this paper, we shall overload this notation for all kinds of other environments we shall be needing, including type environments, and annotated type environments.

The $\lambda^{\sqcup}$-calculus enjoys a number of properties, many of which are what one might expect; we have put these and their proofs in [26].

A *substitution* is a map from variables to terms usually denoted by the letter $\theta$. The application of a substitution $\theta$ to a term $\xi$ is written $\theta\xi$ and replaces all free variables in $\xi$ that are also in the domain of $\theta$ with the corresponding terms they are mapped to. A concrete substitution replacing the variables $\beta_1, \ldots, \beta_n$ with terms $\xi_1, \ldots, \xi_n$ is written $[\xi_1/\beta_1, \ldots, \xi_n/\beta_n]$.

Assuming the usual definitions for the pointwise extension of a lattice $L$, and for monotone (order-preserving) functions between lattices, Figure 2 shows the denotational semantics of $\lambda^{\sqcup}$, where we employ the pointwise lifting of $\cup$ to functions to give semantics to the join of $\lambda^{\sqcup}$. The universe $V_\kappa$ denotes the lattice that is represented by the sort $\kappa$. The base sort $\star$ represents the underlying lattice $\mathcal{L}$ and the function sort $\kappa_1 \Rightarrow \kappa_2$ represents the lattice constructed by pointwise extension of the lattice $V_{\kappa_2}$ restricted to monotone functions.

The denotation function $[\![\cdot]\!]_\rho$ is parameterized with an environment $\rho$ of the given type that provides the values of variables. The denotation of a lambda term is simply an element of the corresponding function space. Applications are therefore mapped directly to the underlying function application of the meta-theory. This is unlike the $\lambda^{\cup}$-calculus of [16] where lambda terms are mapped to singleton sets of functions and function application is defined in terms of the union of the results of individually applying each function. The crucial difference is that we have offloaded this complexity into the definition of the pointwise extension of lattices. It is therefore important to note that the join operator used in the denotation of a term $\xi_1 \sqcup \xi_2$ depends on the sort $\kappa$ of this term and belongs to the lattice $V_\kappa$.

An environment $\rho : \mathbf{AnnVar} \to_{\mathrm{fin}} \bigcup\{V_\kappa \mid \kappa \in \mathbf{AnnSort}\}$ and a sort environment $\Sigma$ are *compatible* if $\mathrm{dom}(\Sigma) = \mathrm{dom}(\rho)$ and for all $\beta \in \mathrm{dom}(\Sigma)$ we have $\rho(\beta) \in V_{\Sigma(\beta)}$. Given two dependency terms $\xi_1$ and $\xi_2$ and a sort $\kappa$ such that $\Sigma \vdash_{\mathrm{s}} \xi_1 : \kappa$ and $\Sigma \vdash_{\mathrm{s}} \xi_2 : \kappa$, we say that $\xi_2$ *subsumes* $\xi_1$ under the environment $\Sigma$, written $\Sigma \vdash_{\mathrm{sub}} \xi_1 \sqsubseteq \xi_2$, if for all environments $\rho$ compatible with $\Sigma$, we have $[\![\xi_1]\!]_\rho \sqsubseteq [\![\xi_2]\!]_\rho$. They are *semantically equal* under $\Sigma$, written $\Sigma \vdash \xi_1 \equiv \xi_2$, if for all environments $\rho$ compatible with $\Sigma$, we have $[\![\xi_1]\!]_\rho = [\![\xi_2]\!]_\rho$.

## 4 The declarative type system

The types and syntax of our source language are given in figure 3. The types of our source language consist of a unit type, and product, sum and function types. As mentioned earlier, let-polymorphism at the type level is not part of the

$$
\begin{array}{rcll}
\tau \in \mathbf{Ty} & ::= & \text{unit} & \text{(unit type)} \\
& | & \tau_1 + \tau_2 & \text{(sum type)} \\
& | & \tau_1 \times \tau_2 & \text{(product type)} \\
& | & \tau_1 \rightarrow \tau_2 & \text{(function type)} \\
t \in \mathbf{Tm} & ::= & x & \text{(variable)} \\
& | & () & \text{(unit constructor)} \\
& | & \lambda x : \tau . t & \text{(abstraction)} \\
& | & t_1 \; t_2 & \text{(application)} \\
& | & (t_1, t_2) & \text{(pair constructor)} \\
& | & \text{proj}_i(t) & \text{(pair projections)} \\
& | & \text{inl}_{\tau_2}(t) \mid \text{inr}_{\tau_1}(t) & \text{(sum constructors)} \\
& | & \textbf{case } t \textbf{ of } \{\text{inl}(x) \rightarrow t_1 ; \text{inr}(y) \rightarrow t_2\} & \text{(sum eliminator)} \\
& | & \mu x : \tau . t & \text{(fixpoint)} \\
& | & \text{seq } t_1 \; t_2 & \text{(forcing)} \\
& | & \text{ann}_\ell(t) & \text{(raise annotation level to } \ell \in \mathcal{L}\text{)}
\end{array}
$$

Fig. 3: The types and terms of the source language type system. The language itself is then hardly suprising and includes variables, a unit constant, lambda abstraction, function application, projection functions for product types, sum constructors, a sum eliminator (case), fixpoints, seq for explicitly forcing evaluation in our call-by-name language, and, finally, a special operation $\text{ann}_\ell(t)$ that raises the annotation level of $t$ to $\ell$. We omit the underlying type system for the source language since it consists mostly of the standard rules (see [26]). A notable exception is the rule for $\text{ann}_\ell(t)$. Such an explicitly annotated term has the same underlying type as $t$:

$$\frac{\Gamma \vdash t : \tau}{\Gamma \vdash \text{ann}_\ell(t) : \tau} \text{[U-ANN]}$$

The annotation $\ell$ imposed on $t$ only becomes relevant in the annotated type system that we discuss next. In the following, we assume the usual definitions for computing the set of free term variables of a term, $\text{ftv}(t)$.

**The annotated type system** The source language is simply a desugared variant of the functional language a programmer deals with. The target language has the same structure, but adds dependency annotations to the source syntax. These annotations are the payload of the dependency analaysis and computed by the algorithm given in section 6, so that the analysis results can be employed in the back-end of a compiler. In other words, the algorithm *elaborates* a source level term into a target term.

The syntax of the target language is shown in figure 4. *Annotated types* of the target language are denoted by $\widehat{\tau}$ and *annotated terms* are denoted by $\widehat{t}$. The annotations that we put on compound types, as well as their components are not just there for uniformity. Because of our non-strict semantics and the

$$
\begin{array}{rcll}
\widehat{\tau} \in \widehat{\mathbf{Ty}} & ::= & \forall \beta :: \kappa.\widehat{\tau} & \text{(annotation quantification)} \\
& | & \widehat{\text{unit}} & \text{(unit type)} \\
& | & \widehat{\tau}_1\langle \xi_1 \rangle + \widehat{\tau}_2\langle \xi_2 \rangle & \text{(sum type)} \\
& | & \widehat{\tau}_1\langle \xi_1 \rangle \times \widehat{\tau}_2\langle \xi_2 \rangle & \text{(product type)} \\
& | & \widehat{\tau}_1\langle \xi_1 \rangle \to \widehat{\tau}_2\langle \xi_2 \rangle & \text{(function type)} \\
\widehat{t} \in \widehat{\mathbf{Tm}} & ::= & \cdots & \\
& | & \lambda x : \widehat{\tau} \,\&\, \xi.\widehat{t} & \text{(abstraction)} \\
& | & \mu x : \widehat{\tau} \,\&\, \xi.\widehat{t} & \text{(fixpoint)} \\
& | & \cdots & \\
& | & \Lambda \beta :: \kappa.\widehat{t} & \text{(dependency abstraction)} \\
& | & \widehat{t}\,\langle \xi \rangle & \text{(dependency application)}
\end{array}
$$

Fig. 4: The annotated types and terms of the target language presence of seq, we can observe the effects on a pair constructor independently of its values, so we have separate annotations to represent these.

On the type level, there is an additional construct $\forall \beta :: \kappa.\widehat{\tau}$ quantifying over an annotation variable $\beta$ of sort $\kappa$. Furthermore, the recursive occurrences in the sum, product and arrow types now each carry an annotation. On the term level, the explicit type annotations of lambda expressions and fixpoints are now annotated types and also include a dependency annotation. Moreover, dependency abstraction and application have been added to reflect the quantification of dependency variables on the type level. We denote the set of free (term) variables in a target term $\widehat{t}$ by $\text{ftv}(\widehat{t})$.

The formal definition of well-formedness for annotated types can be found in [26]. Informally, a type is well-formed only if all annotations are of sort $\star$ and all annotation variables that are used have previously been bound.

Below, we assume the unsurprising recursive definitions for computing the underlying terms $\lfloor \widehat{t} \rfloor$ and underlying types $\lfloor \widehat{\tau} \rfloor$ that correspond to annotated terms $\widehat{t}$ and annotated types $\widehat{\tau}$. We also straightforwardly extend the definition of free annotation variables to annotated types, and denote these by $\text{fav}(\widehat{\tau})$.

**Subtyping** To define subtyping we need an auxiliary relation that says when two annotated types $\widehat{\tau}_1$ and $\widehat{\tau}_2$ *have the same shape*. The unsurprising formal definition is in [26], but essentially they have the same syntactic structure, and in the forall case, quantify over the same annotation variable. It can be quite easily proven that if two types have the same shape, then they have the same underlying type. This is not true the other way around: the annotated types $\forall \beta_1.\forall \beta_2.\text{int}\langle \beta_1 \rangle \to \text{int}\langle \beta_1 \sqcup \beta_2 \rangle$ and $\forall \beta_1.\text{int}\langle \beta_1 \rangle \to \text{int}\langle \beta_1 \rangle$ have the same underlying type, int $\to$ int, but do not have the same shape.

Figure 5 shows the rules defining the subtyping relation on annotated types of the same shape, that allows us to weaken the annotations on a type to a less demanding one. Intuitively, a type $\widehat{\tau}_1$ is a subtype of $\widehat{\tau}_2$ under a sort environment

$$\frac{}{\Sigma \vdash_{\text{sub}} \widehat{\tau} \leqslant \widehat{\tau}}\ [\text{Sub-Refl}]$$

$$\frac{\Sigma \vdash_{\text{sub}} \widehat{\tau}_1 \leqslant \widehat{\tau}_2 \qquad \Sigma \vdash_{\text{sub}} \widehat{\tau}_2 \leqslant \widehat{\tau}_3}{\Sigma \vdash_{\text{sub}} \widehat{\tau}_1 \leqslant \widehat{\tau}_3}\ [\text{Sub-Trans}]$$

$$\frac{\Sigma, \beta :: \kappa \vdash_{\text{sub}} \widehat{\tau}_1 \leqslant \widehat{\tau}_2}{\Sigma \vdash_{\text{sub}} \forall \beta :: \kappa.\widehat{\tau}_1 \leqslant \forall \beta :: \kappa.\widehat{\tau}_2}\ [\text{Sub-Forall}]$$

$$\frac{\begin{array}{cc} \Sigma \vdash_{\text{sub}} \widehat{\tau}_1 \leqslant \widehat{\tau}_1' & \Sigma \vdash_{\text{sub}} \widehat{\tau}_2 \leqslant \widehat{\tau}_2' \\ \Sigma \vdash_{\text{sub}} \xi_1 \sqsubseteq \xi_1' & \Sigma \vdash_{\text{sub}} \xi_2 \sqsubseteq \xi_2' \end{array}}{\Sigma \vdash_{\text{sub}} \widehat{\tau}_1\langle\xi_1\rangle \times \widehat{\tau}_2\langle\xi_2\rangle \leqslant \widehat{\tau}_1'\langle\xi_1'\rangle \times \widehat{\tau}_2'\langle\xi_2'\rangle}\ [\text{Sub-Prod}]$$

$$\frac{\begin{array}{cc} \Sigma \vdash_{\text{sub}} \widehat{\tau}_1' \leqslant \widehat{\tau}_1 & \Sigma \vdash_{\text{sub}} \widehat{\tau}_2 \leqslant \widehat{\tau}_2' \\ \Sigma \vdash_{\text{sub}} \xi_1' \sqsubseteq \xi_1 & \Sigma \vdash_{\text{sub}} \xi_2 \sqsubseteq \xi_2' \end{array}}{\Sigma \vdash_{\text{sub}} \widehat{\tau}_1\langle\xi_1\rangle \to \widehat{\tau}_2\langle\xi_2\rangle \leqslant \widehat{\tau}_1'\langle\xi_1'\rangle \to \widehat{\tau}_2'\langle\xi_2'\rangle}\ [\text{Sub-Arr}]$$

Fig. 5: Subtyping relation ($\Sigma \vdash_{\text{sub}} \widehat{\tau}_1 \leqslant \widehat{\tau}_2$), [Sub-Sum] is like [Sub-Prod]

$\Sigma$, written $\Sigma \vdash_{\text{sub}} \widehat{\tau}_1 \leqslant \widehat{\tau}_2$, if a value of type $\widehat{\tau}_1$ can be used in places where a value of type $\widehat{\tau}_2$ is required. The subtyping relation only relates the annotations inside the types using the subsumption relation $\Sigma \vdash_{\text{sub}} \xi_1 \sqsubseteq \xi_2$ between dependency terms. Moreover, the subtyping relation implicitly demands that both types are well-formed under the environment. The [Sub-Forall] rule requires that the quantified variable has the same name in both types. This is not a restriction, as we can simply rename the variables in one or both of the types accordingly in order to make them match and prevent unintentional capturing of previously free variables. Note that [Sub-Arr] is contravariant for argument positions. We omitted [Sub-Sum] which can be derived from [Sub-Prod] by replacing $\times$ with $+$.

**The annotated type rules** An *annotated type environment* $\widehat{\Gamma}$ is defined analogously to sort environments, but instead maps term variables $x$ to pairs of an annotated type $\widehat{\tau}$ and a dependency term $\xi$. We extend the definition of the set of free annotation variables to annotated environments by taking the union of the free annotation variables of all annotated types and dependency terms occurring in the environment, denoted by $\text{fav}(\widehat{\Gamma})$. We denote the set of annotated type environments by **AnnTyEnv**.

We have now all the definitions in place in order to define the declarative annotated type system shown in figure 6. It consists of judgments of the form $\Sigma \mid \widehat{\Gamma} \vdash_{\text{te}} \widehat{t} : \widehat{\tau}\ \&\ \xi$ expressing that under the sort environment $\Sigma$ and the annotated type environment $\widehat{\Gamma}$, the annotated term $\widehat{t}$ has the annotated type $\widehat{\tau}$ and the dependency term $\xi$. The dependency term in this context is also called the *dependency term* of $\widehat{t}$ [1]. It is implicitly assumed that every type $\widehat{\tau}$ is also well-formed under $\Sigma$, i.e. $\Sigma \vdash_{\mathrm{wft}} \widehat{\tau}$, and that the resulting dependency annotation $\xi$ is of sort $\star$, i.e. $\Sigma \vdash_{\mathrm{s}} \xi : \star$.

We now discuss some of the more interesting rules of figure 6. In [T-Var], both the annotated type and the dependency annotation are looked up in the environment. The dependency annotation of the unit value defaults to the least annotation in [T-Unit]. While we could admit an arbitrary dependency annotation here, the same can be achieved by using the subtyping rule [T-Sub]. We employ this principle more often, e.g., in [T-Abs], and [T-Pair]. This essentially means that the context in which such a term is used completely determines the annotation.

The rule [T-App] may seem overly restrictive by requiring that the types and dependency annotations of the arguments match, and that the dependency annotations of the return value and the function itself are the same. However, in combination with the subtyping rule [T-Sub], this effectively does not restrict the analysis in any way. We see the same happening in other rules, such as [T-Case] and [T-Proj]. Note that the dependency annotation of the argument does not play a role in the resulting dependency annotation of the application. This is because we are dealing with a call by name semantics which means that the argument is not necessarily evaluated before the function call. It should be noted that this does not mean that the dependency annotations of arguments are ignored completely. If the body of a function makes use of an argument, the type system makes sure that its dependency annotation is also incorporated into the result.

When constructing a pair (rule [T-Pair]), the dependency annotations of the components are stored in the type while the pair itself is assigned the least dependency annotation. When accessing a component of a pair (rule [T-Proj]), we require that the dependency annotation of the pair matches the dependency annotation of the projected component. Again, this is no restriction due to the subtyping rule.

In [T-Inl/Inr], the argument to the injection constructor only determines the type and annotation of one component of the sum type while the other component can be chosen arbitrarily as long as the underlying type matches the annotation on the constructor. The destruction of sum types happens in a case statement that is handled by rule [T-Case]. Again, to keep the rule simple and without loss of precision due to judicious use of rule [T-Sub], we may demand that the types of both branches match, and that additionally the dependency annotations of both branches and the scrutinee are equal.

The annotation rule [T-Ann] requires that the dependency annotation of the term being annotated is at least as large as the lattice element $\ell$. In the fixpoint rule, [T-Fix], not only the types but also the dependency annotations of the term itself and the bound variables must match. Note that this rule also

[1] Following the literature of type and effect systems we would much like to use the term "effect" at this point, but decided to use a different term to avoid confusion with the literature on effect handlers.

$$\frac{\widehat{\Gamma}(x) = \widehat{\tau} \mathbin{\&} \xi}{\Sigma \mid \widehat{\Gamma} \vdash_{\mathrm{te}} x : \widehat{\tau} \mathbin{\&} \xi}\ [\text{T-Var}]$$

$$\frac{}{\Sigma \mid \widehat{\Gamma} \vdash_{\mathrm{te}} () : \widehat{\mathrm{unit}} \mathbin{\&} \bot}\ [\text{T-Unit}]$$

$$\frac{\Sigma \mid \widehat{\Gamma}, x : \widehat{\tau}_1 \mathbin{\&} \xi_1 \vdash_{\mathrm{te}} t : \widehat{\tau}_2 \mathbin{\&} \xi_2}{\Sigma \mid \widehat{\Gamma} \vdash_{\mathrm{te}} \lambda x : \widehat{\tau}_1 \mathbin{\&} \xi_1 . t : \widehat{\tau}_1\langle\xi_1\rangle \to \tau_2\langle\xi_2\rangle \mathbin{\&} \bot}\ [\text{T-Abs}]$$

$$\frac{\Sigma \mid \widehat{\Gamma} \vdash_{\mathrm{te}} t_1 : \widehat{\tau}_1\langle\xi_1\rangle \to \widehat{\tau}_2\langle\xi_2\rangle \mathbin{\&} \xi_2 \qquad \Sigma \mid \widehat{\Gamma} \vdash_{\mathrm{te}} t_2 : \widehat{\tau}_1 \mathbin{\&} \xi_1}{\Sigma \mid \widehat{\Gamma} \vdash_{\mathrm{te}} t_1\ t_2 : \widehat{\tau}_2 \mathbin{\&} \xi_2}\ [\text{T-App}]$$

$$\frac{\Sigma \mid \widehat{\Gamma} \vdash_{\mathrm{te}} t_1 : \widehat{\tau}_1 \mathbin{\&} \xi_1 \qquad \Sigma \mid \widehat{\Gamma} \vdash_{\mathrm{te}} t_2 : \widehat{\tau}_2 \mathbin{\&} \xi_2}{\Sigma \mid \widehat{\Gamma} \vdash_{\mathrm{te}} (t_1, t_2) : \widehat{\tau}_1\langle\xi_1\rangle \times \widehat{\tau}_2\langle\xi_2\rangle \mathbin{\&} \bot}\ [\text{T-Pair}]$$

$$\frac{\Sigma \mid \widehat{\Gamma} \vdash_{\mathrm{te}} t : \widehat{\tau}_1\langle\xi_1\rangle \times \widehat{\tau}_2\langle\xi_2\rangle \mathbin{\&} \xi_i}{\Sigma \mid \widehat{\Gamma} \vdash_{\mathrm{te}} \mathrm{proj}_i(t) : \widehat{\tau}_i \mathbin{\&} \xi_i}\ [\text{T-Proj}]$$

$$\frac{\Sigma \mid \widehat{\Gamma} \vdash_{\mathrm{te}} t : \widehat{\tau}_1 \mathbin{\&} \xi_1}{\Sigma \mid \widehat{\Gamma} \vdash_{\mathrm{te}} \mathrm{inl}_{\lfloor\widehat{\tau}_2\rfloor}(t) : \widehat{\tau}_1\langle\xi_1\rangle + \widehat{\tau}_2\langle\xi_2\rangle \mathbin{\&} \bot}\ [\text{T-Inl}]$$

$$\frac{\Sigma \mid \widehat{\Gamma} \vdash_{\mathrm{te}} t : \widehat{\tau}_2 \mathbin{\&} \xi_2}{\Sigma \mid \widehat{\Gamma} \vdash_{\mathrm{te}} \mathrm{inr}_{\lfloor\widehat{\tau}_1\rfloor}(t) : \widehat{\tau}_1\langle\xi_1\rangle + \widehat{\tau}_2\langle\xi_2\rangle \mathbin{\&} \bot}\ [\text{T-Inr}]$$

$$\frac{\Sigma \mid \widehat{\Gamma} \vdash_{\mathrm{te}} t : \widehat{\tau}_1\langle\xi_1\rangle + \widehat{\tau}_2\langle\xi_2\rangle \mathbin{\&} \xi \qquad \begin{array}{c}\Sigma \mid \widehat{\Gamma}, x : \widehat{\tau}_1 \mathbin{\&} \xi_1 \vdash_{\mathrm{te}} t_1 : \widehat{\tau} \mathbin{\&} \xi \\ \Sigma \mid \widehat{\Gamma}, y : \widehat{\tau}_2 \mathbin{\&} \xi_2 \vdash_{\mathrm{te}} t_2 : \widehat{\tau} \mathbin{\&} \xi\end{array}}{\Sigma \mid \widehat{\Gamma} \vdash_{\mathrm{te}} \textbf{case}\ t\ \textbf{of}\ \{\mathrm{inl}(x) \to t_1; \mathrm{inr}(y) \to t_2\} : \widehat{\tau} \mathbin{\&} \xi}\ [\text{T-Case}]$$

$$\frac{\Sigma \mid \widehat{\Gamma} \vdash_{\mathrm{te}} t : \widehat{\tau} \mathbin{\&} \xi \qquad \Sigma \vdash_{\mathrm{sub}} \ell \sqsubseteq \xi}{\Sigma \mid \widehat{\Gamma} \vdash_{\mathrm{te}} \mathrm{ann}_\ell(t) : \widehat{\tau} \mathbin{\&} \xi}\ [\text{T-Ann}]$$

$$\frac{\Sigma \mid \widehat{\Gamma}, x : \widehat{\tau} \mathbin{\&} \xi \vdash_{\mathrm{te}} t : \widehat{\tau} \mathbin{\&} \xi}{\Sigma \mid \widehat{\Gamma} \vdash_{\mathrm{te}} \mu x : \widehat{\tau} \mathbin{\&} \xi . t : \widehat{\tau} \mathbin{\&} \xi}\ [\text{T-Fix}]$$

$$\frac{\Sigma \mid \widehat{\Gamma} \vdash_{\mathrm{te}} t_1 : \widehat{\tau}_1 \mathbin{\&} \xi \qquad \Sigma \mid \widehat{\Gamma} \vdash_{\mathrm{te}} t_2 : \widehat{\tau}_2 \mathbin{\&} \xi}{\Sigma \mid \widehat{\Gamma} \vdash_{\mathrm{te}} \mathrm{seq}\ t_1\ t_2 : \widehat{\tau}_2 \mathbin{\&} \xi}\ [\text{T-Seq}]$$

$$\frac{\Sigma \mid \widehat{\Gamma} \vdash_{\mathrm{te}} t : \widehat{\tau}' \mathbin{\&} \xi' \qquad \Sigma \vdash_{\mathrm{sub}} \widehat{\tau}' \leqslant \widehat{\tau} \qquad \Sigma \vdash_{\mathrm{sub}} \xi' \sqsubseteq \xi}{\Sigma \mid \widehat{\Gamma} \vdash_{\mathrm{te}} t : \widehat{\tau} \mathbin{\&} \xi}\ [\text{T-Sub}]$$

$$\frac{\Sigma, \beta : \kappa \mid \widehat{\Gamma} \vdash_{\mathrm{te}} t : \widehat{\tau} \mathbin{\&} \xi \qquad \beta \notin \mathrm{fav}(\widehat{\Gamma}) \cup \mathrm{fav}(\xi)}{\Sigma \mid \widehat{\Gamma} \vdash_{\mathrm{te}} \Lambda\beta :: \kappa . t : \forall\beta :: \kappa . \widehat{\tau} \mathbin{\&} \xi}\ [\text{T-AnnAbs}]$$

$$\frac{\Sigma \mid \widehat{\Gamma} \vdash_{\mathrm{te}} t : \forall\beta :: \kappa . \widehat{\tau} \mathbin{\&} \xi \qquad \Sigma \vdash_{\mathrm{s}} \xi' : \kappa}{\Sigma \mid \widehat{\Gamma} \vdash_{\mathrm{te}} t\ \langle\xi'\rangle : [\xi' \mathbin{/} \beta]\widehat{\tau} \mathbin{\&} \xi}\ [\text{T-AnnApp}]$$

Fig. 6: Declarative annotated type system ($\Sigma \mid \Gamma \vdash_{\mathrm{te}} \widehat{t} : \widehat{\tau} \mathbin{\&} \xi$)

$$v' \in \mathbf{Nf}' ::= \lambda x : \widehat{\tau}\,\&\,\xi.t \mid \Lambda\beta :: \kappa.t \mid () \mid \mathrm{inl}_\tau(t) \mid \mathrm{inr}_\tau(t) \mid (t_1, t_2)$$
$$v \in \mathbf{Nf} \;\; ::= v' \mid \mathrm{ann}_\ell(v')$$

Fig. 7: Values in the target language admits polyvariant recursion [23], since quantification can occur anywhere in an annotated type. Since seq $t_1$ $t_2$ forces the evaluation of its first argument, it requires that $t_1$'s dependency annotation is part of the final result. This is justified, because the result depends on the termination behavior of $t_1$.

The subtyping rule [T-Sub] allows us to weaken the annotations nested inside a type through the subtyping relation (see figure 5), as well as the dependency annotations itself through the subsumption relation. The rule [T-AnnAbs] introduces an annotation variable $\beta$ of sort $\kappa$ in the body $t$ of the abstraction. The second premise ensures that the annotation variable does not escape its scope determined by the quantification on the type level. The annotation application rule [T-AnnApp] allows the instantiation of an annotation variable with an arbitrary well-sorted dependency term.

## 5 Metatheory

In this section we develop a noninterference proof for our declarative type system, based on a small-step operational call-by-name semantics for the target language.

Figure 7 defines the values of the target language, i.e. those terms that cannot be further evaluated. Apart from a technicality related to annotations, they correspond exactly to the weak head normal forms of terms. The distinction for $\mathbf{Nf}' \subset \mathbf{Nf}$ is made to ensure that there is at most one annotation at top level.

The semantics itself is largely straightforward, except for the handling of annotations. These are moved just as far outwards as necessary in order to reach a normal form, thereby computing the least "permission" an evaluator must possess for computing a certain output. Figure 8 shows two rules: a lifting rule (for applications) and the rule for merging adjacent annotations (see the supplemental material for the others).

In the remainder of this section we state the standard *progress* and *subject reduction* theorems that ensure that our small-step semantics is compatible with

$$\frac{v' \in \mathbf{Nf}'}{(\mathrm{ann}_\ell(v'))\ t_2 \to \mathrm{ann}_\ell(v'\ t_2)}\ [\text{E-LiftApp}]$$

$$\frac{v' \in \mathbf{Nf}'}{\mathrm{ann}_{\ell_1}(\mathrm{ann}_{\ell_2}(v')) \to \mathrm{ann}_{\ell_1 \sqcup \ell_2}(v')}\ [\text{E-JoinAnn}]$$

Fig. 8: Small-step semantics ($t \to t'$) (excerpt)

the annotated type system. The following progress theorem demonstrates that any well-typed term is in normal form, or an evaluation step can be performed.

**Theorem 1 (Progress).** *If* $\emptyset \mid \emptyset \vdash_{\text{te}} t : \widehat{\tau} \,\&\, \xi$*, then either* $t \in \mathbf{Nf}$ *or there is a* $t'$ *such that* $t \to t'$*.*

The subject reduction property says that the reduction of a well-typed term results in a term of the same type.

**Theorem 2 (Subject Reduction).** *If* $\emptyset \mid \emptyset \vdash_{\text{te}} t : \widehat{\tau} \,\&\, \xi$ *and there is a* $t'$ *such that* $t \to t'$*, then* $\emptyset \mid \emptyset \vdash_{\text{te}} t' : \widehat{\tau} \,\&\, \xi$*.*

As expected, subject reduction extends naturally to a sequence of reductions by induction on the length of the reduction sequence:

**Corollary 1.** *If we have* $\emptyset \mid \emptyset \vdash_{\text{te}} t : \widehat{\tau} \,\&\, \xi$ *and* $t \to^* v$*, then* $\emptyset \mid \emptyset \vdash_{\text{te}} v : \widehat{\tau} \,\&\, \xi$*.*

where, as usual, we write $t \to^* v$ if there is a finite sequence of terms $(t_i)_{0 \leqslant i \leqslant n}$ with $t_0 = t$ and $t_n = v \in \mathbf{Nf}$ and reductions $(t_i \to t_{i+1})_{0 \leqslant i < n}$ between them. If there is no such sequence, this is denoted by $t \Uparrow$ and $t$ is said to *diverge*.

Finally, if a term evaluates to an annotated value, this annotation is compatible with the dependency annotation that has been assigned to the term:

**Theorem 3 (Semantic Soundness).** *If we have* $\emptyset \mid \emptyset \vdash_{\text{te}} t : \widehat{\tau} \,\&\, \xi$ *and* $t \to^* \text{ann}_\ell(v')$*, then* $\emptyset \vdash_{\text{sub}} \ell \sqsubseteq \xi$*.*

**The noninterference property** An important theorem for the safety of program transformations/optimizations using the results of dependency analysis is *noninterference*. It guarantees that if there is a target term $t$ depending on some variable $x$ such that $\emptyset \mid x{:}\widehat{\tau}'\&\xi' \vdash_{\text{te}} t{:}\widehat{\tau}\&\xi$ holds and the dependency annotation $\xi'$ of the variable is not encompassed by the resulting dependency annotation $\xi$ (i.e. $\emptyset \vdash_{\text{sub}} \xi' \not\sqsubseteq \xi$), then $t$ will always evaluate to the same normal form, regardless the value of $x$.

Since we are in a non-strict setting, our noninterference property only applies to the topmost constructors of values. This is because the dependency annotations derived in the annotated type system only provide information about the evaluation to weak head normal form. Nested terms might possess lower as well as higher classifications. In particular, the subterms with greater dependency annotations than their enclosing constructors prevent us from making a more general statement because those can still depend on the context whereas the top-level constructor cannot. In the noninterference theorem presented for the SLam calculus, this problem is circumvented by restricting the statement to so called *transparent* types, where the annotations of nested components are decreasing when moving further inward [9].

In the following we consider two normal forms $v_1, v_2 \in \mathbf{Nf}$ to be *similar*, denoted $v_1 \simeq v_2$, if their top level constructors (and annotations, if present) match (see the supplemental material for the unsurprising definition of $\simeq$). So, $v_1 \simeq v_2$ implies that these two values are indistinguishable without further evaluation, which is the property guaranteed by the noninterference theorem.

**Theorem 4 (Noninterference).** *Let $t$ be a target term such that $\emptyset \mid x : \widehat{\tau}' \,\&\, \xi' \vdash_{\mathrm{te}} t : \widehat{\tau} \,\&\, \xi$ and $\emptyset \vdash_{\mathrm{sub}} \xi' \not\sqsubseteq \xi$. Let $v$ be a value.*

*If there is a $t_1$ with $\emptyset \mid \emptyset \vdash_{\mathrm{te}} t_1 : \widehat{\tau}' \,\&\, \xi'$ such that $[t_1 \,/\, x]t \longrightarrow^* v$, then there is a $t'$ such that for all $t_2$ with $\emptyset \mid \emptyset \vdash_{\mathrm{te}} t_2 : \widehat{\tau}' \,\&\, \xi'$ we have $[t_2 \,/\, x]t \longrightarrow^* [t_2 \,/\, x]t'$ and $[t_1 \,/\, x]t' \simeq [t_2 \,/\, x]t'$.*

The noninterference proofs crucially rely on the fact that the source term is well-typed, and the additional assumption $\emptyset \vdash_{\mathrm{sub}} \xi' \not\sqsubseteq \xi$ stating that the dependency annotation of the variable in the context is not encompassed by the dependency annotation of the term being evaluated.

By introducing the restriction to transparent types, we can recover the notion of noninterference used for the SLam calculus. For example, if we have a transparent type $\widehat{\tau}_1\langle\xi_1\rangle \times \widehat{\tau}_2\langle\xi_2\rangle \,\&\, \xi$ (i.e. $\emptyset \vdash_{\mathrm{sub}} \xi_1 \sqsubseteq \xi$ and $\emptyset \vdash_{\mathrm{sub}} \xi_2 \sqsubseteq \xi$) and $\emptyset \vdash_{\mathrm{sub}} \xi' \not\sqsubseteq \xi$ holds, then we also know $\emptyset \vdash_{\mathrm{sub}} \xi' \not\sqsubseteq \xi_1$ and $\emptyset \vdash_{\mathrm{sub}} \xi' \not\sqsubseteq \xi_2$. Otherwise, we would get $\emptyset \vdash_{\mathrm{sub}} \xi' \sqsubseteq \xi$ by transitivity, contradicting the assumption. This means all prerequisites of the noninterference theorem are still fulfilled.

Hence, it is possible in these cases to apply the noninterference theorem to the nested (possibly unevaluated) subterms of a constructor in weak head normal form. As in the work of [1], our noninterference theorem is restricted to deal with terms depending on exactly one variable.

## 6 The type reconstruction algorithm

**Modularity considerations** When designing the type reconstruction algorithm we have two goals: it should be a conservative extension of the underlying type system, and types assigned by the analysis should be as general as possible. Concretely, a function's type must be general enough to be able to adapt to arguments with arbitrary annotations. These two goals give rise to the notion of *fully flexible* and *fully parametric* types defined by [12]. [16] calls these types *conservative* and *pattern* types respectively. Informally, an annotated type is a pattern type if it can be instantiated to any conservative type of the same shape and a conservative type is an analysis of an expression that is able to cope with any arguments it might depend on. These types are conservative in the sense that they make the least assumptions about their arguments and therefore are a conservative estimate compared to other typings with fewer degrees of freedom.

For a pattern type to be instantiable to any conservative type, we first need to make sure that all dependency annotations occurring in it can be instantiated to the corresponding dependency terms in a matching conservative type. This leads to the following definition of a *pattern* in the $\lambda^{\sqcup}$-calculus. It is based on the similar definition by [16] which in turn is a special case of a pattern in higher-order unification theory [4,21]. A $\lambda^{\sqcup}$-term is a *pattern* if it is of the form $f\ \beta_1\ \cdots\ \beta_n$ where $f$ is a free variable and $\beta_1, \ldots, \beta_n$ are distinct bound variables. A unification problem of the form $\forall \beta_1 \cdots \beta_n . f\ \beta_1 \cdots \beta_n = \xi$ where the left-hand side is a pattern is called *pattern unification*. A pattern unification problem $\forall \beta_1 \cdots \beta_n . f\ \beta_1 \cdots \beta_n = \xi$ has a unique most general solution, namely the substitution $[f \mapsto \lambda\beta_1 . \cdots \lambda\beta_n . \xi]$ [4].

$$\frac{\beta \notin \overline{\alpha_i}}{\overline{\alpha_i :: \kappa_{\alpha_i}} \vdash_p \widehat{\text{unit}} \;\&\; \beta\ \overline{\alpha_i} \rhd \beta :: \overline{\kappa_{\alpha_i}} \Rightarrow \star} \text{ [P-UNIT]}$$

$$\frac{\overline{\alpha_i :: \kappa_{\alpha_i}} \vdash_p \widehat{\tau}_1 \;\&\; \xi_1 \rhd \overline{\beta_j :: \kappa_{\beta_j}} \qquad \overline{\alpha_i :: \kappa_{\alpha_i}} \vdash_p \widehat{\tau}_2 \;\&\; \xi_2 \rhd \overline{\gamma_k :: \kappa_{\gamma_k}}}{\overline{\alpha_i :: \kappa_{\alpha_i}} \vdash_p \widehat{\tau}_1\langle\xi_1\rangle \times \widehat{\tau}_2\langle\xi_2\rangle \;\&\; \beta\ \overline{\alpha_i} \rhd \beta :: \overline{\kappa_{\alpha_i}} \Rightarrow \star, \overline{\beta_j :: \kappa_{\beta_j}}, \overline{\gamma_k :: \kappa_{\gamma_k}}} \text{ [P-PROD]}$$

$$\frac{\emptyset \vdash_p \widehat{\tau}_1 \;\&\; \xi_1 \rhd \overline{\beta_j :: \kappa_{\beta_j}} \qquad \overline{\alpha_i :: \kappa_{\alpha_i}}, \overline{\beta_j :: \kappa_{\beta_j}} \vdash_p \widehat{\tau}_2 \;\&\; \xi_2 \rhd \overline{\gamma_k :: \kappa_{\gamma_k}}}{\overline{\alpha_i :: \kappa_{\alpha_i}} \vdash_p \forall \overline{\beta_j :: \kappa_{\beta_j}}.\widehat{\tau}_1\langle\xi_1\rangle \to \widehat{\tau}_2\langle\xi_2\rangle \;\&\; \beta\ \overline{\alpha_i} \rhd \beta :: \overline{\kappa_{\alpha_i}} \Rightarrow \star, \overline{\gamma_k :: \kappa_{\gamma_k}}} \text{ [P-ARR]}$$

Fig. 9: Pattern types ($\Sigma \vdash_p \widehat{\tau} \,\&\, \xi \rhd \Sigma'$), where $\beta \notin \overline{\alpha_i}, \overline{\beta_j}, \overline{\gamma_k}$, and [P-SUM] is like [P-PROD]

The definition of a pattern is then extended to annotated types using the rules from figure 9. Our definition is more precise than the one from previous work in that it makes explicit which variables are expected to be bound and which are free. We require that all variables with different names in the definition of these rules are distinct from each other.

An annotated type and depencency pair $\widehat{\tau} \,\&\, \xi$ is a *pattern type* under the sort environment $\Sigma$ if the judgment $\Sigma \vdash_p \widehat{\tau} \,\&\, \xi \rhd \Sigma'$ holds for some $\Sigma'$. We call the variables in $\Sigma$ *argument variables* and the variables in $\Sigma'$ *pattern variables.*

*Example 1.* A simple pattern type with the pattern variables $\beta :: \star \Rightarrow \star$ and $\beta' :: \star \Rightarrow \star \Rightarrow \star$ is

$$\forall \beta_1 :: \star.\widehat{\text{unit}}\langle\beta_1\rangle \to (\forall \beta_2 :: \star.\widehat{\text{unit}}\langle\beta_2\rangle \to \widehat{\text{unit}}\langle\beta'\ \beta_1\ \beta_2\rangle)\langle\beta\ \beta_1\rangle$$

Note that since $\beta_1$ is quantified on the function arrow chain, it is passed on to the second function arrow. However, it is not propagated into the second argument. In general, annotations on the return type may depend on the annotations of all previous arguments while annotations of the arguments may not. This prevents any dependency between the annotations of arguments and guarantees that they are as permissive as possible. This is also why pattern variables in a covariant position are passed on to the next higher level while pattern variables in arguments are quantified in the enclosing function arrow. This allows the caller of a function to instantiate the dependency annotations of the parameters to the actual arguments.

As we stated earlier, a conservative function type makes the least assumptions over its arguments. Formally, this means that arguments of conservative functions are pattern types. We will later see that a pattern type can be instantiated to any conservative type of the same shape. On the other hand, non-functional conservative types are not constrained in their annotations. These characteristics are captured by the following definition based on *conservative types* [16] and *fully flexible types* [12].

An annotated type $\widehat{\tau}$ is *conservative* if

$$\frac{\beta \text{ fresh}}{\overline{\alpha_i :: \kappa_{\alpha_i}} \vdash_c \text{unit} : \widehat{\text{unit}} \,\&\, \beta\ \overline{\alpha_i} \rhd \beta :: \overline{\kappa_{\alpha_i}} \Rightarrow \star}\ [\text{C-Unit}]$$

$$\frac{\overline{\alpha_i :: \kappa_{\alpha_i}} \vdash_c \tau_1 : \widehat{\tau}_1 \,\&\, \xi_1 \rhd \overline{\beta_j :: \kappa_{\beta_j}} \qquad \overline{\alpha_i :: \kappa_{\alpha_i}} \vdash_c \tau_2 : \widehat{\tau}_2 \,\&\, \xi_2 \rhd \overline{\gamma_k :: \kappa_{\gamma_k}}}{\overline{\alpha_i :: \kappa_{\alpha_i}} \vdash_c \tau_1 \times \tau_2 : \widehat{\tau}_1\langle\xi_1\rangle \times \widehat{\tau}_2\langle\xi_2\rangle \,\&\, \beta\ \overline{\alpha_i} \rhd \beta :: \overline{\kappa_{\alpha_i}} \Rightarrow \star, \overline{\beta_j :: \kappa_{\beta_j}}, \overline{\gamma_k :: \kappa_{\gamma_k}}}\ [\text{C-Prod}]$$

$$\frac{\emptyset \vdash_c \tau_1 : \widehat{\tau}_1 \,\&\, \xi_1 \rhd \overline{\beta_j :: \kappa_{\beta_j}} \qquad \overline{\alpha_i :: \kappa_{\alpha_i}}, \overline{\beta_j :: \kappa_{\beta_j}} \vdash_c \tau_2 : \widehat{\tau}_2 \,\&\, \xi_2 \rhd \overline{\gamma_k :: \kappa_{\gamma_k}}}{\overline{\alpha_i :: \kappa_{\alpha_i}} \vdash_c \tau_1 \to \tau_2 : \forall \overline{\beta_j :: \kappa_{\beta_j}}.\widehat{\tau}_1\langle\xi_1\rangle \to \widehat{\tau}_2\langle\xi_2\rangle \,\&\, \beta\ \overline{\alpha_i} \rhd \beta :: \overline{\kappa_{\alpha_i}} \Rightarrow \star, \overline{\gamma_k :: \kappa_{\gamma_k}}}\ [\text{C-Arr}]$$

Fig. 10: Type completion ($\Sigma \vdash_c \tau{:}\widehat{\tau}\&\xi\rhd\Sigma'$), all $\beta$ fresh, [C-Sum] is like [C-Prod]

1. $\widehat{\tau} = \widehat{\text{unit}}$, or
2. $\widehat{\tau} = \widehat{\tau}_1\langle\xi_1\rangle + \widehat{\tau}_2\langle\xi_2\rangle$ and both $\widehat{\tau}_1$ and $\widehat{\tau}_2$ are conservative, or
3. $\widehat{\tau} = \widehat{\tau}_1\langle\xi_1\rangle \times \widehat{\tau}_2\langle\xi_2\rangle$ and both $\widehat{\tau}_1$ and $\widehat{\tau}_2$ are conservative, or
4. $\widehat{\tau} = \forall\overline{\beta_j :: \kappa_j}.\widehat{\tau}_1\langle\xi_1\rangle \to \widehat{\tau}_2\langle\xi_2\rangle$ and both (a) $\emptyset \vdash_p \widehat{\tau}_1 \,\&\, \xi_1 \rhd \overline{\beta_j :: \kappa_j}$ and (b) $\widehat{\tau}_2$ is conservative.

Moreover, an annotated type and depencency pair $\widehat{\tau} \,\&\, \xi$ is *conservative* if $\widehat{\tau}$ is *conservative* and an annotated type environment $\widehat{\Gamma}$ is *conservative* if for all $x \in \text{dom}(\widehat{\Gamma})$, $\widehat{\Gamma}(x)$ is conservative.

The following type signature for the function $f$ is a conservative type that takes the function type from example 1 as an argument.

$$\begin{aligned} f : \forall\beta :: \star \Rightarrow \star.\forall\beta' :: \star \Rightarrow \star \Rightarrow \star.\forall\beta_3 :: \star.& \\ (\forall\beta_1 :: \star.\widehat{\text{unit}}\langle\beta_1\rangle \to (\forall\beta_2 :: \star.\widehat{\text{unit}}\langle\beta_2\rangle \to \widehat{\text{unit}}\langle\beta'\ \beta_1\ \beta_2\rangle)\langle\beta\ \beta_1\rangle)\langle\beta_3\rangle& \\ \to \widehat{\text{unit}}\langle\beta_3 \sqcup \beta\ \bot \sqcup \beta'\ \bot\ \ell\rangle \,\&\, \bot& \end{aligned}$$

Note that the pattern variables of the argument have been bound in the top-level function type. This allows callers of $f$ to instantiate these patterns.

We can extend the previous definition of pattern types to the type completion relation shown in figure 10. It relates every underlying type $\tau$ with a pattern type $\widehat{\tau}$ such that $\widehat{\tau}$ erases to $\tau$. It is defined through judgments $\Sigma \vdash_c \tau : \widehat{\tau} \,\&\, \xi \rhd \Sigma'$ with the meaning that under the sort environment $\Sigma$, $\tau$ is completed to the annotated type $\widehat{\tau}$ and the dependency annotation $\xi$ containing the pattern variables $\Sigma'$. The completion relation can also be interpreted as a function taking $\Sigma$ and $\tau$ as arguments and returning $\widehat{\tau}$, $\xi$ and $\Sigma'$.

Lastly, we revisit the examples from the previous sections and show how a pattern type can be mechanically derived from an underlying type.

In example 1 we presented a pattern type for the underlying type unit $\to$ unit $\to$ unit. Using the type completion relation, we can derive the pattern type,

$$(\forall\beta_1.\widehat{\text{unit}}\langle\beta_1\rangle \to (\forall\beta_2.\widehat{\text{unit}}\langle\beta_2\rangle \to \widehat{\text{unit}}\langle\beta'\ \beta_1\ \beta_2\rangle)\langle\beta\ \beta_1\rangle) \,\&\, \beta_3$$

without having to guess. This is because the components $\widehat{\tau}$, $\xi$ and $\Sigma'$ in a judgment $\Sigma \vdash_c \tau : \widehat{\tau} \,\&\, \xi \rhd \Sigma'$ are uniquely determined by $\Sigma$ and $\tau$ from looking at the syntax alone. The resulting pattern type contains three pattern variables, $\beta :: \star \Rightarrow \star$, $\beta' :: \star \Rightarrow \star \Rightarrow \star$ and $\beta_3 :: \star$. If the initial sort environment is empty, these are also the only free variables of the pattern type.

Based on the type completion relation we can define least type completions. These are conservative types that are subtypes of all other conservative types of the same shape. Therefore, all annotations occurring in positive positions on the top level function arrow chain must also be least. We do not need to consider arguments here because those are by definition equal up to alpha-conversion due to being pattern types. We define the *least annotation term* of sort $\kappa$ as

$$\begin{aligned} \bot_\star &= \bot \\ \bot_{\kappa_1 \Rightarrow \kappa_2} &= \lambda\beta : \kappa_1 . \bot_{\kappa_2} . \end{aligned}$$

These least annotation terms correspond to the least elements of our bounded lattice for a given sort $\kappa$. This in turn leads us to the definition of the least completion of type $\tau$ (see figure 10) by substituting all free variables in the completion with the least annotation of the corresponding sort, i.e.

$$\bot_\tau = [\overline{\bot_{\kappa_i} \;/\; \beta_i}]\widehat{\tau} \text{ for } \emptyset \vdash_c \tau : \widehat{\tau} \;\&\; \xi \rhd \overline{\beta_i :: \kappa_i}.$$

**The algorithm** We can now move on to the type reconstruction algorithm that performs the actual analysis. At its core lies algorithm $\mathcal{R}$ shown in figure 11. The input of the algorithm is a triple $(\widehat{\Gamma}, \Sigma, t)$ consisting of a well-typed source term $t$, an annotated type environment $\widehat{\Gamma}$ providing the types and dependency annotations of the free term variables in $t$ and a sort environment $\Sigma$ mapping each free annotation variable in scope to its sort. It returns a triple $\widehat{t} : \widehat{\tau} \;\&\; \xi$ consisting of an elaborated term $\widehat{t}$ in the target language (that erases to the source term $t$), an annotated type $\widehat{\tau}$ and an dependency annotation $\xi$ such that $\Sigma \mid \widehat{\Gamma} \vdash_{\text{te}} \widehat{t} : \widehat{\tau} \;\&\; \xi$ holds. In the definition of $\mathcal{R}$, to avoid clutter, we write $\Gamma$ instead of $\widehat{\Gamma}$ because we are only dealing with one kind of type environment.

The algorithm relies on the invariant that all types in the type environment and the inferred type must be conservative. In the version of [16], all inferred dependency annotations (including those nested as annotations in types) had to be canonically ordered as well. But as it turned out that this canonically ordered form was not enough for deciding semantic equality, so we lifted this requirement. We still mark those places in the algorithm where canonicalization would have occurred with $\llbracket \cdot \rrbracket$., but the actual result of this operation does not matter as long as the dependency terms remain equivalent.

The algorithm for computing the least upper bound of types ($\sqcup$ in figure 12) requires that both types are conservative, have the same shape and use the same names for bound variables. The latter can be ensured by $\alpha$-conversion while the former two requirements are fulfilled by how this function is used in $\mathcal{R}$.

The restriction to conservative types allows us to ignore functions arguments because these are always required to be pattern types, which are unique up to $\alpha$-equivalence. This alleviates the need for computing a corresponding greatest lower bound of types, because the algorithm only traverses covariant positions.

$$\mathcal{R} : \mathbf{AnnTyEnv} \times \mathbf{SortEnv} \times \mathbf{Tm} \rightarrow \widehat{\mathbf{Tm}} \times \widehat{\mathbf{Ty}} \times \mathbf{AnnTm}$$

$$
\begin{array}{l}
\mathcal{R}(\Gamma; \Sigma; x) = x : \Gamma(x) \\
\mathcal{R}(\Gamma; \Sigma; ()) = () : \widehat{\mathrm{unit}} \mathbin{\&} \bot \\
\mathcal{R}(\Gamma; \Sigma; \mathrm{ann}_\ell(t)) = \\
\quad \mathbf{let}\ \widehat{t} : \widehat{\tau} \mathbin{\&} \xi = \mathcal{R}(\Gamma; \Sigma; t) \\
\quad \mathbf{in}\ \mathrm{ann}_\ell(\widehat{t}) : \widehat{\tau} \mathbin{\&} \llbracket \xi \sqcup \ell \rrbracket_\Sigma \\
\mathcal{R}(\Gamma; \Sigma; \mathrm{seq}\ t_1\ t_2) = \\
\quad \mathbf{let}\ \widehat{t_1} : \widehat{\tau}_1 \mathbin{\&} \xi_1 = \mathcal{R}(\Gamma; \Sigma; t_1) \\
\quad \phantom{\mathbf{let}}\ \widehat{t_2} : \widehat{\tau}_2 \mathbin{\&} \xi_2 = \mathcal{R}(\Gamma; \Sigma; t_2) \\
\quad \mathbf{in}\ \mathrm{seq}\ \widehat{t_1}\ \widehat{t_2} : \widehat{\tau}_2 \mathbin{\&} \llbracket \xi_1 \sqcup \xi_2 \rrbracket_\Sigma \\
\mathcal{R}(\Gamma; \Sigma; (t_1, t_2)) = \\
\quad \mathbf{let}\ \widehat{t_1} : \widehat{\tau}_1 \mathbin{\&} \xi_1 = \mathcal{R}(\Gamma; \Sigma; t_1) \\
\quad \phantom{\mathbf{let}}\ \widehat{t_2} : \widehat{\tau}_2 \mathbin{\&} \xi_2 = \mathcal{R}(\Gamma; \Sigma; t_2) \\
\quad \mathbf{in}\ (\widehat{t_1}, \widehat{t_2}) : \widehat{\tau}_1\langle \xi_1 \rangle \times \widehat{\tau}_2\langle \xi_2 \rangle \mathbin{\&} \bot \\
\mathcal{R}(\Gamma; \Sigma; \mathrm{inl}_{\tau_2}(t)) = \\
\quad \mathbf{let}\ \widehat{t} : \widehat{\tau}_1 \mathbin{\&} \xi_1 = \mathcal{R}(\Gamma; \Sigma; t) \\
\quad \mathbf{in}\ \mathrm{inl}_{\tau_2}(\widehat{t}) : \widehat{\tau}_1\langle \xi_1 \rangle + \bot_{\tau_2}\langle \bot \rangle \mathbin{\&} \bot \\
\mathcal{R}(\Gamma; \Sigma; \mathrm{inr}_{\tau_1}(t)) = \\
\quad \mathbf{let}\ \widehat{t} : \widehat{\tau}_2 \mathbin{\&} \xi_2 = \mathcal{R}(\Gamma; \Sigma; t) \\
\quad \mathbf{in}\ \mathrm{inr}_{\tau_1}(\widehat{t}) : \bot_{\tau_1}\langle \bot \rangle + \widehat{\tau}_2\langle \xi_2 \rangle \mathbin{\&} \bot \\
\mathcal{R}(\Gamma; \Sigma;\ \mathbf{case}\ t_1\ \mathbf{of}\ \{\mathrm{inl}(x) \rightarrow t_2; \\
\qquad\qquad\qquad\qquad\quad \mathrm{inr}(y) \rightarrow t_3\}) = \\
\quad \mathbf{let}\ \widehat{t_1} : \widehat{\tau}\langle \xi \rangle + \widehat{\tau}'\langle \xi' \rangle \mathbin{\&} \xi_1 = \mathcal{R}(\Gamma; \Sigma; t_1) \\
\quad \phantom{\mathbf{let}}\ \widehat{t_2} : \widehat{\tau}_2 \mathbin{\&} \xi_2 = \mathcal{R}(\Gamma, x : \widehat{\tau} \mathbin{\&} \xi; \Sigma; t_2) \\
\quad \phantom{\mathbf{let}}\ \widehat{t_3} : \widehat{\tau}_3 \mathbin{\&} \xi_3 = \mathcal{R}(\Gamma, y : \widehat{\tau}' \mathbin{\&} \xi'; \Sigma; t_3) \\
\quad \mathbf{in}\ \mathbf{case}\ \widehat{t_1}\ \mathbf{of}\ \{\mathrm{inl}(x) \rightarrow \widehat{t_2}; \mathrm{inr}(y) \rightarrow \widehat{t_3}\} \\
\qquad\qquad : \llbracket \widehat{\tau}_2 \sqcup \widehat{\tau}_3 \rrbracket_\Sigma \mathbin{\&} \llbracket \xi_1 \sqcup \xi_2 \sqcup \xi_3 \rrbracket_\Sigma
\end{array}
$$

$$
\begin{array}{l}
\mathcal{R}(\Gamma; \Sigma; \mathrm{proj}_i(t)) = \\
\quad \mathbf{let}\ \widehat{t} : \widehat{\tau}_1\langle \xi_1 \rangle \times \widehat{\tau}_2\langle \xi_2 \rangle \mathbin{\&} \xi = \mathcal{R}(\Gamma; \Sigma; t) \\
\quad \mathbf{in}\ \mathrm{proj}_i(\widehat{t}) : \widehat{\tau}_i \mathbin{\&} \llbracket \xi \sqcup \xi_i \rrbracket_\Sigma \\
\mathcal{R}(\Gamma; \Sigma; \lambda x : \tau_1.t) = \\
\quad \mathbf{let}\ \widehat{\tau}_1 \mathbin{\&} \beta \rhd \overline{\beta_i :: \kappa_i} = \mathcal{C}([\,]; \tau_1) \\
\quad \phantom{\mathbf{let}}\ \Gamma' = \Gamma, x : \widehat{\tau}_1 \mathbin{\&} \beta \\
\quad \phantom{\mathbf{let}}\ \Sigma' = \Sigma, \overline{\beta_i :: \kappa_i} \\
\quad \phantom{\mathbf{let}}\ \widehat{t} : \widehat{\tau}_2 \mathbin{\&} \xi_2 = \mathcal{R}(\Gamma'; \Sigma'; t) \\
\quad \mathbf{in}\ \ \Lambda\overline{\beta_i :: \kappa_i}.\lambda x : \widehat{\tau}_1 \mathbin{\&} \beta.\widehat{t} \\
\qquad\qquad : \forall \overline{\beta_i :: \kappa_i}.\widehat{\tau}_1\langle \beta \rangle \rightarrow \widehat{\tau}_2\langle \xi_2 \rangle \mathbin{\&} \bot \\
\mathcal{R}(\Gamma; \Sigma; t_1\ t_2) = \\
\quad \mathbf{let}\ \widehat{t_1} : \widehat{\tau}_1 \mathbin{\&} \xi_1 = \mathcal{R}(\Gamma; \Sigma; t_1) \\
\quad \phantom{\mathbf{let}}\ \widehat{t_2} : \widehat{\tau}_2 \mathbin{\&} \xi_2 = \mathcal{R}(\Gamma; \Sigma; t_2) \\
\quad \phantom{\mathbf{let}}\ \widehat{\tau}_2'\langle \beta \rangle \rightarrow \widehat{\tau}\langle \xi \rangle \rhd \overline{\beta_i} = \mathcal{I}(\widehat{\tau}_1) \\
\quad \phantom{\mathbf{let}}\ \theta = [\beta \mapsto \xi_2] \circ \mathcal{M}([\,]; \widehat{\tau}_2'; \widehat{\tau}_2) \\
\quad \mathbf{in}\ \widehat{t_1}\ \langle \overline{\theta\beta_i} \rangle\ \widehat{t_2} : \llbracket \theta\widehat{\tau} \rrbracket_\Sigma \mathbin{\&} \llbracket \xi_1 \sqcup \theta\xi \rrbracket_\Sigma \\
\mathcal{R}(\Gamma; \Sigma; \mu x : \tau.t) = \\
\quad \mathbf{do}\ i; \widehat{\tau}_0 \mathbin{\&} \xi_0 \leftarrow 0; \bot_\tau \mathbin{\&} \bot \\
\qquad \mathbf{repeat}\ \widehat{t}_{i+1} : \widehat{\tau}_{i+1} \mathbin{\&} \xi_{i+1} \\
\qquad\qquad\qquad \leftarrow \mathcal{R}(\Gamma, x : \widehat{\tau}_i \mathbin{\&} \xi_i; \Sigma; t) \\
\qquad\qquad\quad i \leftarrow i + 1 \\
\qquad \mathbf{until}\quad (\widehat{\tau}_{i-1} \equiv \widehat{\tau}_i \wedge \xi_{i-1} \equiv \xi_i) \\
\qquad \mathbf{return}\ (\mu x : \widehat{\tau}_i \mathbin{\&} \xi_i.\widehat{t}_i) : \widehat{\tau}_i \mathbin{\&} \xi_i
\end{array}
$$

Fig. 11: Type reconstruction algorithm ($\mathcal{R}$)

The handling of $\lambda$-abstractions uses the type completion algorithm $\mathcal{C}$ of figure 12, that defers its work to the type completion relation defined earlier which can be interpreted in a functional way (see figure 10). The underlying type of the function argument is completed to a pattern type. The function body is analyzed in the presence of the newly introduced pattern variables. Note that this pattern type is also conservative, thereby preserving the invariant that the context only holds conservative types. The inferred annotated type of the lambda abstraction universally quantifies over all pattern variables and the quantification is reflected on the term level through annotation abstractions $\Lambda\beta :: \kappa.t$.

In order to analyze function applications, we need two more auxiliary algorithms. The first one is the instantiation procedure $\mathcal{I}$ (see figure 12) which instantiates all top-level quantifiers with fresh annotation variables. The second is the matching algorithm $\mathcal{M}$ (see figure 12) which instantiates a pattern type

$$\begin{array}{lll}
\sqcup : \widehat{\mathbf{Ty}} \times \widehat{\mathbf{Ty}} \to \widehat{\mathbf{Ty}} & & \\
\widehat{\text{unit}} & \sqcup\, \widehat{\text{unit}} & = \widehat{\text{unit}} \\
(\widehat{\tau}_1\langle\xi_1\rangle \times \widehat{\tau}_2\langle\xi_2\rangle) & \sqcup\, (\widehat{\tau}_1'\langle\xi_1'\rangle \times \widehat{\tau}_2'\langle\xi_2'\rangle) & = (\widehat{\tau}_1 \sqcup \widehat{\tau}_1')\langle\xi_1 \sqcup \xi_1'\rangle \times (\widehat{\tau}_2 \sqcup \widehat{\tau}_2')\langle\xi_2 \sqcup \xi_2'\rangle \\
(\widehat{\tau}_1\langle\beta\rangle \to \widehat{\tau}_2\langle\xi_2\rangle) & \sqcup\, (\widehat{\tau}_1\langle\beta\rangle \to \widehat{\tau}_2'\langle\xi_2'\rangle) & = \widehat{\tau}_1\langle\beta\rangle \to (\widehat{\tau}_2 \sqcup \widehat{\tau}_2')\langle\xi_2 \sqcup \xi_2'\rangle \\
(\forall\beta :: \kappa.\widehat{\tau}) & \sqcup\, (\forall\beta :: \kappa.\widehat{\tau}') & = \forall\beta :: \kappa.\widehat{\tau} \sqcup \widehat{\tau}'
\end{array}$$

$$\begin{array}{l}
\mathcal{C} : \mathbf{SortEnv} \to \widehat{\mathbf{Ty}} \times \mathbf{AnnTm} \times \mathbf{SortEnv} \\
\mathcal{C}(\Sigma;\tau) = \widehat{\tau}\;\&\;\xi \rhd \overline{\beta_i :: \kappa_i}\ \textbf{where}\ \Sigma \vdash_c \tau : \widehat{\tau}\;\&\;\xi \rhd \overline{\beta_i :: \kappa_i}
\end{array}$$

$$\begin{array}{ll}
\mathcal{I} : \widehat{\mathbf{Ty}} \to \widehat{\mathbf{Ty}} \times \mathbf{SortEnv} & \\
\mathcal{I}(\forall\beta :: \kappa.\widehat{\tau}) & = \textbf{let}\ \widehat{\tau}' \rhd \Sigma = \mathcal{I}(\widehat{\tau})\ \textbf{in}\ [\beta \mapsto \beta'](\widehat{\tau}') \rhd \beta' :: \kappa, \Sigma\ \textbf{where}\ \beta'\ \textit{be fresh} \\
\mathcal{I}(\widehat{\tau}) & = \widehat{\tau} \rhd [\,]
\end{array}$$

$$\begin{array}{l}
\mathcal{M} : \mathbf{SortEnv} \times \widehat{\mathbf{Ty}} \times \widehat{\mathbf{Ty}} \to \mathbf{AnnSubst} \\
\mathcal{M}(\Sigma; \widehat{\text{unit}};\quad \widehat{\text{unit}}) \quad = [\,] \\
\mathcal{M}(\Sigma; \widehat{\tau}_1'\langle\beta\,\overline{\beta_i}\rangle \times \widehat{\tau}_2'\langle\beta'\,\overline{\beta_i}\rangle; \widehat{\tau}_1\langle\xi_1\rangle \times \widehat{\tau}_2\langle\xi_2\rangle) = \\
\quad [\beta \mapsto \lambda\overline{\beta_i :: \Sigma(\beta_i)}.\xi_1, \beta' \mapsto \lambda\overline{\beta_i :: \Sigma(\beta_i)}.\xi_2] \circ \mathcal{M}(\Sigma; \widehat{\tau}_1'; \widehat{\tau}_1) \circ \mathcal{M}(\Sigma; \widehat{\tau}_2'; \widehat{\tau}_2) \\
\mathcal{M}(\Sigma; \widehat{\tau}_1\langle\beta\rangle \to \widehat{\tau}_2'\langle\beta'\,\overline{\beta_i}\rangle;\quad \widehat{\tau}_1\langle\beta\rangle \to \widehat{\tau}_2\langle\xi\rangle) \ = \\
\quad [\beta' \mapsto \lambda\overline{\beta_i :: \Sigma(\beta_i)}.\xi] \circ \mathcal{M}(\Sigma; \widehat{\tau}_2'; \widehat{\tau}_2) \\
\mathcal{M}(\Sigma; \forall\beta :: \kappa.\widehat{\tau}'; \forall\beta :: \kappa.\widehat{\tau}) = \mathcal{M}(\Sigma, \beta :: \kappa; \widehat{\tau}'; \widehat{\tau})
\end{array}$$

Fig. 12: Least upper bound of types ($\sqcup$), completion ($\mathcal{C}$), instantiation ($\mathcal{I}$), and matching ($\mathcal{M}$). Rules for $\cdot + \cdot$ in $\sqcup$ and $\mathcal{M}$ are like those for $\cdot \times \cdot$.

with a conservative type of the same shape. It returns a substitution obtained by performing pattern unification on corresponding annotations.

**Soundness and Completeness** An annotated type environment $\widehat{\Gamma}$ is well-formed under an environment $\Sigma$, if $\widehat{\Gamma}$ is conservative and for all bindings $x : \widehat{\tau}\;\&\;\xi$ in $\widehat{\Gamma}$ we have $\Sigma \vdash_{\text{wft}} \widehat{\tau}$ and $\Sigma \vdash_{\text{s}} \xi : \star$.

In order to demonstrate the correctness of the reconstruction algorithm presented in this section we have to show that for every well-typed underlying term, it produces an analysis (i.e. annotated types and dependency annotations) that can be derived in the annotated type system (see figure 6). That is to say, algorithm $\mathcal{R}$ is sound w.r.t. the annotated type system.

**Theorem 5.** *Let $t$ be a source term, $\Sigma$ a sort environment and $\widehat{\Gamma}$ an annotated type environment well-formed under $\Sigma$ such that $\mathcal{R}(\widehat{\Gamma}; \Sigma; t) = \widehat{t} : \widehat{\tau}\;\&\;\xi$ for some $\widehat{t}$, $\widehat{\tau}$ and $\xi$.*

*Then, $\Sigma \mid \widehat{\Gamma} \vdash_{\text{te}} \widehat{t} : \widehat{\tau}\;\&\;\xi$, $\Sigma \vdash_{\text{wft}} \widehat{\tau}$, $\Sigma \vdash_{\text{s}} \xi : \star$ and $\widehat{\tau}$ is conservative.*

The next step is to show that our analysis succeeds in deriving an annotated type and dependency annotation for any well-typed source term: it is *complete*.

The crucial part here is the termination of the fixpoint iteration. In order to show the convergence of the fixpoint iteration, we start by defining an equivalence relation on annotated type and depencency pairs.

Our type reconstruction algorithm handles polymorphic recursion through Kleene-Mycroft-iteration. Such an algorithm is based on fixpoint iteration and needs a way to decide whether two dependency terms are equal according to the denotational semantics of $\lambda^{\sqcup}$.

A straightforward way to decide semantic equivalence is to enumerate all possible environments and compare the denotations of the two terms in all of these (possibly after some semantics preserving normalization). This only works if the dependency lattice $\mathcal{L}$ is finite.

For some analyses, e.g., the set of all program locations in a slicing analysis, $\mathcal{L} = V_\star$ is finite but large, and deciding equality in this fashion becomes impractical. To alleviate this problem, our prototype implementation applies a partial canonicalization procedure which, while not complete, can serve as an approximation of equality: if two canonicalized dependency terms become syntactically equal, then we can be assured that they are semantically equal, but if they are not we can still apply the above procedure to the canonicalized dependency terms. We omit formal details from the paper.

We can now state our completeness results for the type reconstruction algorithm. Here, we write $\Gamma \vdash_{\mathrm{t}} t : \tau$ to say that term $t$ has type $\tau$ under the environment $\Gamma$ in the underlying type system.

**Theorem 6 (Completeness).** *Given a source term $t$, a sort environment $\Sigma$, an annotated type environment $\widehat{\Gamma}$ well-formed under $\Sigma$, and an underlying type $\tau$ such that $\lfloor\widehat{\Gamma}\rfloor \vdash_{\mathrm{t}} t : \tau$, then there are $\widehat{t}$, $\widehat{\tau}$ and $\xi$ such that $\mathcal{R}(\widehat{\Gamma}; \Sigma; t) = \widehat{t} : \widehat{\tau} \,\&\, \xi$ and $\lfloor\widehat{\tau}\rfloor = \tau$, $\lfloor\widehat{t}\rfloor = t$.*

As a corollary of the foregoing theorems, our analysis is a conservative extension of the underlying type system.

**Corollary 2 (Conservative Extension).** *Let $t$ be a source term, $\tau$ be a type and $\Gamma$ a type environment such that $\Gamma \vdash_{\mathrm{t}} t : \tau$. Then there are $\Sigma$, $\widehat{\Gamma}$, $\widehat{t}$, $\widehat{\tau}$, $\xi$ such that $\Sigma \mid \widehat{\Gamma} \vdash_{\mathrm{te}} \widehat{t} : \widehat{\tau} \,\&\, \xi$ with $\lfloor\widehat{t}\rfloor = t$, $\lfloor\widehat{\tau}\rfloor = \tau$ and $\lfloor\widehat{\Gamma}\rfloor = \Gamma$.*

## 7 Implementation and Examples

Beyond the definition of the annotated system and the development of the associated algorithm and meta-theory we also have a REPL prototype implementation of our analysis in Haskell. Compared to the annotated type system in the paper, the prototype provides support for booleans and integers, including literals and conditionals **if** $c$ **then** $t_1$ **else** $t_2$ for which the type rules can be straightforwardly derived. Concrete lattice implementations are provided only for binding-time analysis and security analysis, but the reconstruction algorithm abstracts away from the choice for a particular lattice, so it is easy to add new instances. The implementation is available at http://www.staff.science.uu.nl/~hage0101/

prototype-hrp.zip. Below we walk through a few examples, taking advantage of the slightly extended source language that our implementation supports. More (detailed) examples are discussed in [26].

**Construction and Elimination** Whenever something is constructed, be it a product, a sum or a lambda abstraction, the outermost dependency annotation is $\bot$. This is because the analysis aims to produce the best possible and thereby least annotations for a given source program.

Consider the case of binding-time analysis, and suppose we have a variable of function type $f : \forall\beta.\mathrm{int}\langle\beta\rangle \rightarrow \mathrm{int}\langle\beta\rangle \,\&\, \mathbf{D}$. We can see that it preserves the annotations of its arguments, i.e. if we apply $f$ to a static value, the return annotation is also instantiated to be static. The function itself, however, is dynamic. And therefore, the whole result of the function application must also be dynamic, because we cannot know which particular function has been assigned to $f$.

As elimination always introduces a dependency in the program, and this can uncover subtleties arising when functions only differ in their termination behavior. For example, compare $\lambda p : \mathrm{int} \times \mathrm{int}.p$ with $\lambda p : \mathrm{int} \times \mathrm{int}.(\mathrm{proj}_1(p), \mathrm{proj}_2(p))$. In a call-by-value language, these two functions would be (extensionally) equivalent. However, with non-strict evaluation, $p$ might be a non-terminating computation. In that case, applying the former function would diverge, while the latter function at least produces the pair constructor. This is also reflected in the annotated types that are inferred. For the former, we get

$$\forall\beta_0, \beta_1, \beta_2 :: \star.(\mathrm{int}\langle\beta_0\rangle \times \mathrm{int}\langle\beta_1\rangle)\langle\beta_2\rangle \rightarrow (\mathrm{int}\langle\beta_0\rangle \times \mathrm{int}\langle\beta_1\rangle)\langle\beta_2\rangle \,\&\, \mathbf{S}, \text{and}$$

$$\forall\beta_0, \beta_1, \beta_2 :: \star.(\mathrm{int}\langle\beta_0\rangle \times \mathrm{int}\langle\beta_1\rangle)\langle\beta_2\rangle \rightarrow (\mathrm{int}\langle\beta_0 \sqcup \beta_2\rangle \times \mathrm{int}\langle\beta_1 \sqcup \beta_2\rangle)\langle\mathbf{S}\rangle \,\&\, \mathbf{S}$$

for the latter. In particular, the annotation of the product in the second type signature is $\mathbf{S}$. Therefore, it can not depend on the input of the function.

**Polymorphic Recursion** One class of functions where the analysis benefits from polymorphic recursion are those that permute their arguments on recursive calls. Our example is a slightly modified version of an example from [5]:

$$\mu f : \mathrm{bool} \rightarrow \mathrm{bool} \rightarrow \mathrm{bool}.\lambda x : \mathrm{bool}.\lambda y : \mathrm{bool}.\ \mathbf{if}\ x\ \mathbf{then}\ \mathit{true}\ \mathbf{else}\ f\ y\ x$$

In an analysis with monomorphic recursion, the analysis assigns the same annotation to both parameters, large enough to accommodate for both arguments. This is due to the permutation of the arguments in the else branch. An analysis with polymorphic recursion is allowed to use a different instantiation for $f$ in that case. Our algorithm hence infers the following most general type.

$$\forall\beta_1 :: \star.\widehat{\mathrm{bool}}\langle\beta_1\rangle \rightarrow (\forall\beta_2 :: \star.\widehat{\mathrm{bool}}\langle\beta_2\rangle \rightarrow \widehat{\mathrm{bool}}\langle\beta_1 \sqcup \beta_2\rangle)\langle\bot\rangle \,\&\, \bot$$

We see that the result of the function indeed depends on the annotations of both arguments, as both end up in the condition of the if-expression at some point. Yet, both arguments are completely unrestricted, and unrelated in their annotations. In contrast, a type system with monomorphic recursion would only admit a weaker type, possibly similar to

$$\forall \beta_1 :: \star.\widehat{\text{bool}}\langle\beta_1\rangle \to (\widehat{\text{bool}}\langle\beta_1\rangle \to \widehat{\text{bool}}\langle\beta_1\rangle)\langle\bot\rangle \ \&\ \bot$$

A real world example of this kind is Euclid's algorithm for computing the greatest common divisor(see [26]).

**Higher-Ranked Polyvariance** This section discusses several examples for the dependency analysis instance of binding time analysis, comparing our outcomes with a let-polyvariant analysis[29].

A simple example to start with is a function that applies a function to both components of a pair[2]

$$\begin{aligned}&\mathit{both} : (\text{int} \to \text{int}) \to \text{int} \times \text{int} \to \text{int} \times \text{int}\\&\mathit{both} = \lambda f : \text{int} \to \text{int}.\lambda p : \text{int} \times \text{int}.(f\ (\text{proj}_1(p)), f\ (\text{proj}_2(p)))\end{aligned}$$

Suppose in the context of binding-time analysis that *both* is used to apply a statically known function to a pair whose first component is always computable at compile time, but whose second component is dynamic. For simplicity's sake, the function is the identity on integers.

$$\begin{aligned}&\mathit{id} : \text{int} \to \text{int}\\&\mathit{id} = \lambda x : \text{int}.x\end{aligned}$$

A non-higher-ranked analysis would assign types to *both* and *id*. The annotation on the function argument to *both* must be large enough to accommodate both components of the pair as input. When we consider the call *both id p* for some pair $p{:}\text{int}\langle\mathbf{S}\rangle \times \text{int}\langle\mathbf{D}\rangle \& \mathbf{S}$. Then, the whole call has the type $\text{int}\langle\mathbf{D}\rangle \times \text{int}\langle\mathbf{D}\rangle$.

Our higher-ranked analysis infers the following conservative types for *id* and *both*.

$$\begin{aligned}&\mathit{id} : \forall\beta :: \star.\text{int}\langle\beta\rangle \to \text{int}\langle\beta\rangle \ \&\ \bot\\&\mathit{id} = \Lambda\beta :: \star.\lambda x : \text{int} \ \&\ \beta.x\end{aligned}$$

$$\begin{aligned}&\mathit{both} : \forall\beta_1 :: \star.\forall\beta_2 :: \star \Rightarrow \star.(\forall\beta :: \star.\text{int}\langle\beta\rangle \to \text{int}\langle\beta_2\ \beta\rangle)\langle\beta_1\rangle\\&\quad \to (\forall\beta_3,\beta_4,\beta_5 :: \star.(\text{int}\langle\beta_3\rangle \times \text{int}\langle\beta_4\rangle)\langle\beta_5\rangle\\&\quad \to (\text{int}\langle\beta_2\ (\beta_3 \sqcup \beta_5) \sqcup \beta_1\rangle \times \text{int}\langle\beta_2\ (\beta_4 \sqcup \beta_5) \sqcup \beta_1\rangle)\langle\mathbf{S}\rangle)\langle\mathbf{S}\rangle \ \&\ \mathbf{S}\\&\mathit{both} = \Lambda\beta_1 :: \star.\Lambda\beta_2 :: \star \Rightarrow \star.\lambda f : (\forall\beta :: \star.\text{int}\langle\beta\rangle \to \text{int}\langle\beta_2\ \beta\rangle).\\&\quad \Lambda\beta_3 :: \star.\Lambda\beta_4 :: \star.\Lambda\beta_5 :: \star.\lambda p : \text{int}\langle\beta_3\rangle \times \text{int}\langle\beta_4\rangle.\\&\qquad (f\ \langle\beta_3 \sqcup \beta_5\rangle\ (\text{proj}_1(p)), f\ \langle\beta_4 \sqcup \beta_5\rangle\ (\text{proj}_2(p)))\end{aligned}$$

In case of *both*, the function parameter $f$ can be instantiated separately for each component because our analysis assigns it a type that universally quantifies over

[2] NB. *both* is a simplified instance of a traversal $\forall f.\mathit{Applicative}\ f \Rightarrow (\mathit{Int} \to f\ \mathit{Int}) \to (\mathit{Int}, \mathit{Int}) \to f\ (\mathit{Int}, \mathit{Int})$, in order to fit the restrictions of the source language [6,15].

the annotation of its argument. It is evident from the type signature that the components of the resulting pair only depend on the corresponding components of the input pair, and the function and the input pair itself. They do not depend on the respective other component of the input.

If we again consider the call *both id p*, we obtain $\beta_2 = \lambda\beta :: \star.\beta$, $\beta_1 = \beta_3 = \beta_5 = \mathbf{S}$ and $\beta_4 = \mathbf{D}$ through pattern unification. Normalization of the resulting dependency terms results in the expected return type $\text{int}\langle\mathbf{S}\rangle \times \text{int}\langle\mathbf{D}\rangle$.

The generality provided by the higher-ranked analysis extends to an arbitrarily deep nesting of function arrows. The following example demonstrates this for two levels of arrows. Functions with more than two levels of arrows can arise directly in actual programs, but even more so in desugared code, e.g., when type classes in Haskell are implemented via explicit dictionary passing. Due to limitations of our source language, the examples are syntactically heavily restricted.

Consider the following function that takes a function argument which again requires a function.

$$\begin{aligned}
&\mathit{foo} : ((\text{int} \to \text{int}) \to \text{int}) \to \text{int} \times \text{int}\\
&\mathit{foo} = \lambda f : (\text{int} \to \text{int}) \to \text{int}.(f\ (\lambda x : \text{int}.x), f\ (\lambda x : \text{int}.0))
\end{aligned}$$

The higher-ranked analysis infers the following type and target term (where we omitted the type in the argument of the lambda term because it essentially repeats what is already visible in the top level type signature).

$$\begin{aligned}
&\mathit{foo} : \forall\beta_4 :: \star.\forall\beta_3 :: \star \Rightarrow (\star \Rightarrow \star) \Rightarrow \star.\\
&\quad(\forall\beta_2 :: \star.\forall\beta_1 :: \star \Rightarrow \star.(\forall\beta_0 :: \star.\text{int}\langle\beta_0\rangle \to \text{int}\langle\beta_1\ \beta_0\rangle)\langle\beta_2\rangle\\
&\quad\ \to \text{int}\langle\beta_3\ \beta_2\ \beta_1\rangle)\langle\beta_4\rangle\\
&\quad\ \to (\text{int}\langle\beta_3\ \mathbf{S}\ (\lambda\beta_5 :: \star.\beta_5) \sqcup \beta_4\rangle \times \text{int}\langle\beta_3\ \mathbf{S}\ (\lambda\beta_6 :: \star.\mathbf{S}) \sqcup \beta_4\rangle)\langle\mathbf{S}\rangle\ \&\ \mathbf{S}\\
&\mathit{foo} = \Lambda\beta_4 :: \star.\Lambda\beta_3 :: \star \Rightarrow (\star \Rightarrow \star) \Rightarrow \star.\lambda f : \cdots .\\
&\quad(f\ \langle\mathbf{S}\rangle\ \langle\lambda\beta_0 :: \star.\beta_0\rangle\ (\Lambda\beta_5 :: \star.\lambda x : \text{int}\ \&\ \beta_5.x)\\
&\quad,f\ \langle\mathbf{S}\rangle\ \langle\lambda\beta_0 :: \star.\mathbf{S}\rangle\ \ (\Lambda\beta_6 :: \star.\lambda x : \text{int}\ \&\ \beta_6.1))
\end{aligned}$$

Since the type of $f$ is a pattern type, the argument to $f$ is also a pattern type by definition. Therefore, the analysis of $f$ depends on the analysis of the function passed to it. This gives rise to the *higher-order effect operator* $\beta_3$ [12]. Thus, $f$ can be applied to any function with a conservative type of the right shape. As our algorithm always infers conservative types, the type of $f$ is as general as possible. This is reflected in the body of the lambda where in both cases $f$ is instantiated with the dependency annotation corresponding to the function passed to it. The result of this instantiation can be observed in the returned product type where $\beta_3$ is applied to the effect operators $\lambda\beta_0 :: \star.\beta_0$ and $\lambda\beta_0 :: \star.\mathbf{S}$ corresponding to the respective functions used as arguments to $f$.

Only when we finally apply *foo*, the resulting annotations can be evaluated.

$$\begin{aligned}
&\mathit{bar} : \forall\alpha_2 :: \star.\forall\alpha_1 :: \star \Rightarrow \star.(\forall\alpha_0 :: \star.\text{int}\langle\alpha_0\rangle \to \text{int}\langle\alpha_1\ \alpha_0\rangle)\langle\alpha_2\rangle\\
&\qquad\qquad\qquad\qquad\qquad\quad \to \text{int}\langle\alpha_1\ \mathbf{D} \sqcup \alpha_2\rangle\ \&\ \mathbf{S}\\
&\mathit{bar} = \Lambda\alpha_2 :: \star.\Lambda\alpha_1 :: \star \Rightarrow \star.\lambda f : \cdots .f\ (\text{ann}_{\mathbf{D}}(0))
\end{aligned}$$

For *bar* we obtain $foo\ bar : \text{int}\langle\mathbf{D}\rangle \times \text{int}\langle\mathbf{S}\rangle\ \&\ \mathbf{S}$. In this case, $\beta_3 = \lambda\beta_2 :: \star.\lambda\beta_1 :: \star \Rightarrow \star.\beta_1\ \mathbf{D} \sqcup \beta_2$, because *bar* applies its argument to a value with dynamic binding time. This causes the first component of the returned pair to be deemed dynamic as well. On the other hand, in the second component *bar* is applied to a constant function. Thus, regardless of the argument's dynamic binding time, the resulting binding time is static. In a rank-1 system we would get $\text{int}\langle\mathbf{D}\rangle \times \text{int}\langle\mathbf{D}\rangle$ instead of $\text{int}\langle\mathbf{D}\rangle \times \text{int}\langle\mathbf{S}\rangle$.

## 8 Related Work

The basis for most type systems of functional programming languages is the Hindley-Milner type system [22]. Our algorithm $R$ strongly resembles the well-known type inference algorithm for the Hindley-Milner type system, *Algorithm W* [3], a distinct advantage of our approach. The idea to define an annotated type system as a means to design static analyses for higher-order languages is attributed to [19]. The major technical difference compared to a let-polyvariant analysis is that our annotations form a simply typed lambda-calculus.

Full reconstruction for a higher-ranked polyvariant annotated type system was first considered by [12] in the context of a control-flow analysis. However, we found that the (constraint-based) algorithm as presented in [12] generates constraints free of cycles. Therefore, it cannot faithfully reflect the constraints necessary for the fixpoint combinator. The algorithm incorrectly concludes for the following example that only the first and third 'False' term flow into the condition $x$, but not the second one.

$$(\mathit{fix}\ (\lambda f.\,\lambda x.\,\lambda y.\,\lambda z.\,\mathbf{if}\ x\ \mathbf{then}\ \mathit{True}\ \mathbf{else}\ f\ z\ x\ y))\ \mathit{False}\ \mathit{False}\ \mathit{False}$$

We reproduced this mistake with their implementation and verified that the mistake was not a simple bug in that implementation.

Close to our formulation is the (unpublished) work of [16] which deals with exception analysis, which uses a simply typed lambda-calculus with sets to represent annotations. We have chosen a more modular approach in which we offload much of the complexity of dealing with lattice values to the lattice. In [16] terms from the simply typed lambda-calculus with sets are canonicalized and then checked for alpha equivalence during Kleene-Mycroft iteration. We found however that two terms can have different canonical forms even though they are actually semantically equivalent. This causes Koot's reconstruction algorithm to diverge on a particular class of programs, because the inferred annotations continue to grow. The simplest such program we found is the following.

$$\mu f : (\text{unit} \to \text{unit}) \to \text{unit} \to \text{unit}.\lambda g : \text{unit} \to \text{unit}.\lambda x : \text{unit}.g\ (f\ g\ x)$$

Our solution is to apply canonicalization to simplify terms as much as possible, and then compare the outcomes for all possible inputs.

The Dependency Core Calculus was introduced by [1] as a unifying framework for dependency analyses. Instances include binding-time analysis (see, e.g.,

[29]), exception analysis [17,16], secure information flow analysis [9] and static slicing [27]. They devised the *Dependency Core Calculus* (DCC) to which each instance of a dependency analysis can be mapped. This allowed them to compare different dependency analyses, uncover problems with existing instance analyses and to simplify proofs of noninterference [8,20]. The instance analyses in [1] were defined as a monovariant type and effect system with subtyping, for a monomorphic call-by-name language. An implicit, let-polymorphic implementation of DCC, FlowCaml, was developed by [25]. It is not higher-ranked.

The difference between DCC and our analysis is to a large extent a different focus: the DCC is a calculus defined in a way that any calculus that elaborates to DCC has the noninterference property and any other properties proven for the calculus. On the other hand, our analysis is meant to be implemented in a compiler (with the added precision), and that implementation (and its associated meta-theory) can then be reused inside the compiler for a variety of analyses. Comparable to DCC, we have proven a noninterference property for our generic higher-rank polyvariant dependency analysis, so that all its instances inherit it.

The Haskell community supports an implementation of DCC in which the (security) annotations are lifted to the Haskell type level [2]. Since the GHC compiler supports higher-rank types, the code written with this library can in fact model security flows with higher-rank. Because of the general undecidability of full reconstruction for higher-rank types [14], the programmer must however provide explicit type information. In [18], the authors introduce dependent flow types, that allows them to express a large variety of security policies. An essential difference with our work is that our approach is fully automated.

Early on in our research, we observed that the approach of [11] may lead to similar precision gains as higher-ranked annotations do. Since they deal with a different analysis, a direct comparison is impossible to make at this time.

## 9 Conclusion and Future Work

We have defined a higher-rank annotation polymorphic type system for a generic dependency analysis, established its soundness and provided a sound and complete reconstruction algorithm. Examples show that we can achieve higher precision than plain let-polyvariance. The analysis we have defined is for a call-by-name language. We expect the results to hold as well for a lazy language, but chose call-by-name for reduced bookkeeping in the proofs. We also believe the analysis can be adapted relatively easily to one for a call-by-value language, by letting the annotation on the argument flow into the effect of the call. However, we would need to re-examine the metatheory.

In future work we want to consider whether we can further refine the canonicalization of $\lambda^{\sqcup}$ terms so that syntactic equality up to alpha-equivalence can completely replace our current approach.

**Acknowledgments** We acknowledge the contributions of Ruud Koot in unpublished work that made this work possible.
